# Synthetic Biodegradable Aliphatic Polyester Nanocomposites Reinforced with Nanohydroxyapatite and/or Graphene Oxide for Bone Tissue Engineering Applications

**DOI:** 10.3390/nano9040590

**Published:** 2019-04-10

**Authors:** Yuchao Li, Chengzhu Liao, Sie Chin Tjong

**Affiliations:** 1 Department of Materials Science and Engineering, Liaocheng University, Liaocheng 252059, China; 2 Department of Materials Science and Engineering, Southern University of Science and Technology, Shenzhen 518055, China; liaocz@sustech.edu.cn; 3 Department of Physics, City University of Hong Kong, Tat Chee Avenue, Kowloon, Hong Kong, China

**Keywords:** scaffold, bionanocomposite, nanohydroxyapatite, graphene oxide, aliphatic polyesters, osteoblast, mineralization, tissue engineering, additive manufacturing, fused deposition modeling

## Abstract

This paper provides review updates on the current development of bionanocomposites with polymeric matrices consisting of synthetic biodegradable aliphatic polyesters reinforced with nanohydroxyaptite (nHA) and/or graphene oxide (GO) nanofillers for bone tissue engineering applications. Biodegradable aliphatic polyesters include poly(lactic acid) (PLA), polycaprolactone (PCL) and copolymers of PLA-PGA (PLGA). Those bionanocomposites have been explored for making 3D porous scaffolds for the repair of bone defects since nHA and GO enhance their bioactivity and biocompatibility by promoting biomineralization, bone cell adhesion, proliferation and differentiation, thus facilitating new bone tissue formation upon implantation. The incorporation of nHA or GO into aliphatic polyester scaffolds also improves their mechanical strength greatly, especially hybrid GO/nHA nanofilllers. Those mechanically strong nanocomposite scaffolds can support and promote cell attachment for tissue growth. Porous scaffolds fabricated from conventional porogen leaching, and thermally induced phase separation have many drawbacks inducing the use of organic solvents, poor control of pore shape and pore interconnectivity, while electrospinning mats exhibit small pores that limit cell infiltration and tissue ingrowth. Recent advancement of 3D additive manufacturing allows the production of aliphatic polyester nanocomposite scaffolds with precisely controlled pore geometries and large pores for the cell attachment, growth, and differentiation in vitro, and the new bone formation in vivo.

## 1. Introduction

**Figure 1:**
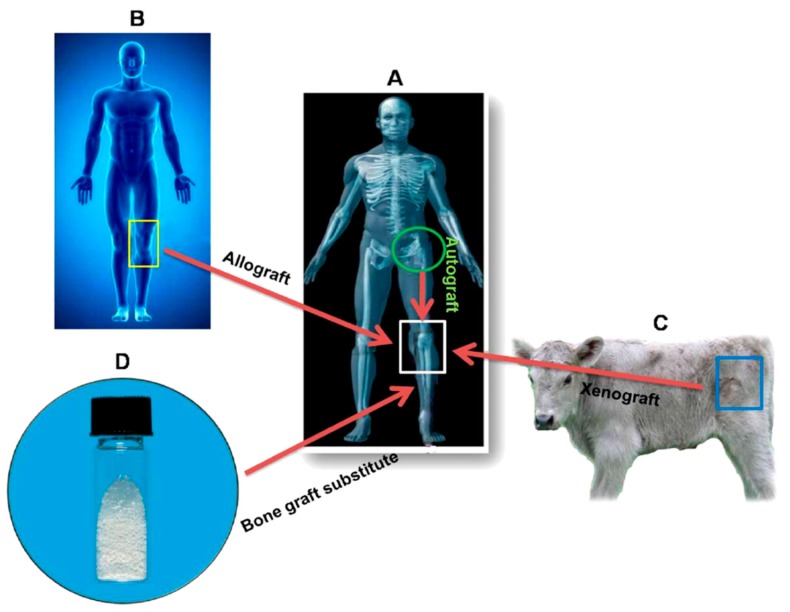
Schematic showing types of bone grafts to be implanted into human body: (**A**) autograft, (**B**) allograft, (**C**) xenograft and (**D**) synthetic bone graft substitute. Reproduced from [8], published by BioMed Central Ltd. under the Creative Commons Attribution license.

Recently, bone defects and disorders as well as related diseases have attracted considerable public health concerns due to an increase in the ageing trauma population, bone tumor, injuries from sports activities and traffic accidents [1,2,3,4,5,6,7]. With the ageing population and longer life expectancy, osteoporosis is increasingly becoming a global health issue. The number of elderly people is rising at a rapid rate in the Asia-Pacific region, Europe and North America. Osteoporosis results from a reduction in the bone strength and density, leading to an increased risk of fragility fractures in elderly [2,3]. Moreover, cancer patients also suffer from osteoporosis due to accelerated loss of bone mass by chemotherapy and the use of drugs [4,5]. Therefore, osteoporosis is a bone disease having a high impact on morbidity and mortality [5]. Thus, the demand for the use of bone grafts increases globally, and surgeons use autografts, allografts or xenografts to treat patients with serious bone injuries (Figure 1) [8]. Autografts removed from the bones of patients provide osteoinductive, osteoconductive, and osteogenic properties [9]. However, autografts suffer from the donor site pain, inflammation and morbidity, and complications following harvest of bone grafts. Allografts and xenografts carry the risk of immunological rejection and possible disease transmission. In this respect, bone tissue engineering especially the design of synthetic bone graft substitutes and 3D porous scaffolds, has been shown to be effective in inducing repair and reconstruction of bone defects, and for eliminating risk of disease transmission [8,10].

In bone tissue engineering, porous scaffolds serve as an artificial extracellular matrix (ECM) to provide structural and mechanical support for bone cells to attach, spread, multiply, and differentiate, thereby acting as a template to grow cell for bone tissue regeneration [11,12,13,14]. The scaffolds for bone tissue engineering must be osteoinductive, osteoconductive, and osteointegrative. These properties permit strong integration of the scaffolds with the host tissue upon transplantation Scaffolds are designed to match the mechanical properties of human trabecular bones. In general, compressive modulus and compressive strength of trabecular bones depend greatly on the wet/dry conditions, density, porosity and anatomic location of the bones [15]. Moreover, scaffolds must be three-dimensional (3D), highly porous with pore sizes ≥100 µm and highly interconnected pore structure to promote bone ingrowth, nutrient transport and metabolic waste removal [16]. Porous 3D scaffolds with or without cells and growth factors such as bone morphogenetic protein-2 (BMP-2), transforming growth factor-β (TGF-β), are then transplanted into bone defective sites [17]. For a nude scaffold, cells will recruit in vivo from the host tissue. Metals and their alloys are often employed to repair bone fractures because of their outstanding mechanical properties. However, elastic modulus of stainless steels and Ti-based alloys typically used for biomedical applications is much higher than that of human bones, leading to the so-called ‘stress shielding’ effect [18]. Moreover, metals always suffer from the corrosion issues [19,20]. Corrosion and released ions may induce inflammation response, cell apoptosis and the foreign body reaction. Magnesium and its alloys with mechanical properties close to those of human cortical bones find useful applications as the bone fixation devices and scaffolds [21]. They are degradable in physiological environment. However, it is difficult to accurately control the pore morphologies of Mg scaffolds with good mechanical strength. In addition, high corrosion rate of Mg-based materials and the rise of pH in surrounding tissues due to released Mg ions tend to limit their application as biodegradable scaffolds in orthopedics [22,23]. 

Polymers with good processability and light weight are ideal materials for biomedical applications [24,25,26]. Polymers can be synthesized and tailored to meet specific needs and applications through controlling their chemical compositions and structures. As such, they find attractive biomedical applications in tissue engineering, therapeutics and drug delivery [8,27,28]. From the literature, non-degradable polyetheretherketone (PEEK) with high temperature durability, excellent radiation stability and high stiffness has been designed to make scaffolds for interbody spinal fusion [29]. However, poor degradation behavior of PEEK limits its further application in bone tissue engineering. By contrast, degradable polymers including natural and synthetic are typically used for bone tissue engineering applications. Several natural polymers such as collagen, chitosan, gelatin, alginate, and synthetic polymers like aliphatic polyesters and hydrophilic polyvinyl alcohol have been employed for these purposes [16,28,30]. Biodegradable aliphatic polyesters include poly(lactic acid) (PLA), poly(glycolic acid) (PGA), polycaprolactone (PCL) and copolymers of PLA-PGA (PLGA). Collagen is the most widely adapted into scaffolds as it constitutes an organic phase of the bone tissue. Collagen can be extracted from animal (bovine and porcine skin/bone) and marine sources [31,32]. However, animal-derived collagen may pose a risk to public health and safety because of the outbreak of infectious livestock diseases recently, e.g., bovine spongiform encephalopathy and African swine fever. In addition, collagen and other natural polymers also suffer from low mechanical strength, inferior thermal stability and processability, and poor control over the degradation rate. Thus, synthetic polymers offer many advantages over natural polymers for making bone scaffolds including tailored degradation rate and higher mechanical strength [16]. 

Bone tissue is a biocomposite consisting of a collagen fibrils and hydroxyapatite nanorods [33]. Hydroxyapatite (Ca_10_(PO_4_)_6_(OH)_2_) (HA) is a brittle material with low fracture toughness. This precludes its use as a standalone biomaterial in orthopedics. HA has a calcium-to-phosphate ratio of 1.67, and shows excellent osteoconductivity due to its chemical and structural similarity to inorganic components of human bones and teeth. Thus, it is beneficial to incorporate HA into polymers to form biocomposites with improved osteoblast adhesion and proliferation. For instance, Bonfield and coworkers first introduced 40 vol% HA microparticles (mHA) into high-density polyethylene (HDPE) for use in non-load-bearing maxillofacial applications [34]. Synthetic aliphatic polyesters have poor cellular affinity and low bone bioactivity, so it is beneficial to incorporate HA into those polymers to overcome these limitations. The combination of degradable polyesters and inorganic bioactive mHA have been studied by several research groups [35,36,37]. Russias et al. reported that PLA-based composites with 70–85 wt% mHA exhibit mechanical properties that match closely with those of human cortical bones [35]. Apparently, conventional polymer biocomposites require very high mHA loadings for promoting bone cell adhesion and proliferation. This is a typical behavior of traditional polymer microcomposites [38,39,40]. 

Recent progress in nanotechnology has opened a new route in the development of advanced functional nanomaterials for industrial and biomedical applications [41,42,43,44,45,46,47]. In the latter case, nanoceramics based on alumina, titania and hydroxyapatite have been reported to enhance bone growth due to their large surface area over their micro-grained materials [48]. Moreover, nanoceramic surfaces mimic microenvironments of the natural bones, thereby promoting protein adsorption and stimulating new bone formation. In particular, nanocrystalline HA (nHA) favors large amounts adsorption of vitronectin and BMP-2 on its surface, thus accelerating new bone formation effectively [48,49,50]. Therefore, bioactivity and biocompatibility of the polymers, especially biodegradable aliphatic polyesters, can be significantly improved by adding nHA fillers [51,52,53,54,55,56,57,58,59,60,61]. 

Graphene is a two-dimensional layer of sp^2^-bonded carbon atoms that are arranged in a hexagonal lattice. It is a building block for zero-dimensional (0D) bucky ball and graphene quantum dot (GQD), one-dimensional (1D) carbon nanotube, and three-dimensional (3D) graphite. Graphene exhibits exceptionally high elastic modulus of 1 TPa, high electrical mobility of 2 × 10^5^ cm^2^ V^−1^ s^−1^, and excellent light transparency of 97.7% [62,63,64]. Graphene and its derivatives can be fabricated through several processing routes, leading to the products with fundamentally different structural, chemical and physical properties. Graphene prepared from chemical vapor deposition (CVD) is mainly used for optoelectronic applications [42]. In contrast, graphene oxide (GO) prepared from chemical oxidation of graphite flakes in strong oxidants, and reduced graphene oxide (rGO) obtained by chemical or thermal reduction of GO, are excellent candidates for biomedical applications [65,66,67,68]. Generally, insulating GO exhibits a much lower elastic modulus of 207.6 ± 23.4 GPa comparing with pristine graphene [69]. Nevertheless, the stiffness of GO is much higher than that of nHA (110 GPa) [70]. Thus, very low GO filler contents are needed for enhancing mechanical strength and biocompatibility of aliphatic polyesters [71,72,73,74,75,76,77,78,79,80]. Very recently, Tjong and coworkers demonstrated that PLA hybrid nanocomposites reinforced with both nHA and GO fillers exhibit better compatibility than binary PLA/nHA composites [81]. This is because graphene and its derivatives promote new bone formation by facilitating osteoblastic adhesion and growth, and osteogenic differentiation of mesenchymal stem cells (MSCs) on their surfaces [82,83,84,85]. An additive effect between nHA and GO nanofillers leads to further enhancement in the mechanical strength and biocompatibility of PLA. This article gives the state-of-the art review on the recent development, mechanical performance, in vitro biodegradation, mineralization and cell cultivation, and in vivo animal models of biodegradable aliphatic polyesters reinforced with nHA, and/or GO nanofillers for bone tissue engineering applications, especially in the past four years.

## 2. Preparation of Nanofillers

### 2.1. Nanohydroxyapatite

Several synthetic strategies have been employed for fabricating nHA including wet chemical precipitation, hydrothermal method, microwave irradiation, sol gel, emulsion, etc. The morphology, crystallite size, stoichiometry and chemical composition of nHA prepared from these routes differ substantially from one to another [86,87,88,89,90,91,92,93,94]. Among these, wet chemical precipitation is one of the most commonly technique used for forming nHA due to its simplicity, and ease of fabrication without using organic solvents. The precipitation process only requires inexpensive Ca and P chemical reagents. Typical Ca precursors include Ca(OH)_2_, CaCl_2_, and Ca(NO_3_)_2_, while P precursors are H_3_PO_4_, Na_2_HPO_4_ and (NH_4_)_2_HPO_4_. Representative chemical reactions for precipitating nHA are given as follows [86]:

10 Ca(OH)_2_ + 6 H_3_PO_4_ → Ca_10_(PO4)_6_(OH)_2_ + 18 H_2_O (1)

10 Ca(NO_3_)_2_ + 6 (NH_4_)_2_HPO_4_ + 2 H_2_O → Ca_10_(PO4)_6_(OH)_2_ + 8 HNO_3_ (2)

**Figure 2:**
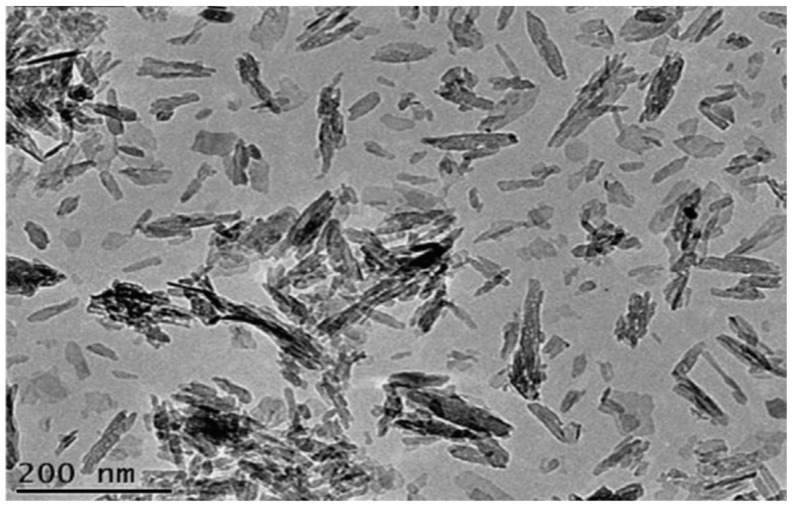
Transmission electron microscope image of nanohydroxyapatite prepared from wet chemical precipitation process [88]. Reproduced with permission from Elsevier, published by Elsevier 2015.

**Figure 3:**
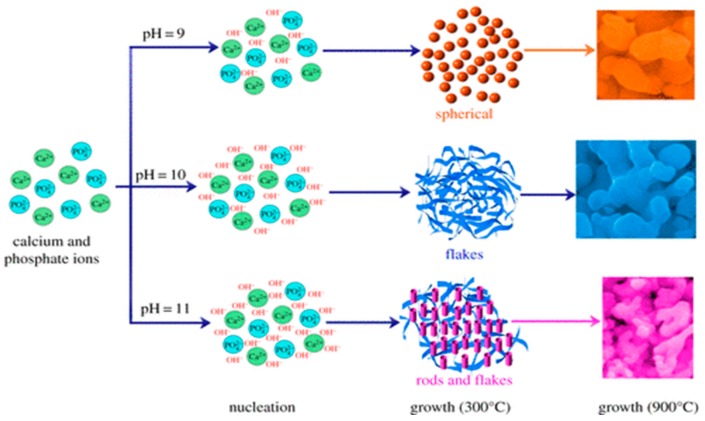
Effects of the solution pH values (9, 10 and 11) and sintering temperatures (300 °C and 900 °C) on the morphologies of nHA. Reproduced from [89]. Published by the Royal Society under the Creative Commons Attribution license (http://creativecommons.org/licenses/by/4.0/).

The composition and morphology of nHA products depend largely on the selected precursors and processing parameters or conditions. Ramesh et al. synthesized nHA with needle-like morphologies by using Ca(OH)_2_ and H_3_PO_4_ precursors; the pH of solution was controlled at 10.5 through the addition of ammonium solution (Figure 2) [88]. Very recently, Rodrıguez-Lugo et al. demonstrated that the pH of aqueous solution and sintering temperature play important roles in controlling the final shape of nHA products (Figure 3). They employed calcium nitrate and ammonium phosphate precursors as well as ammonium hydroxide (NH_4_OH) to synthesize nHA of different morphologies [89]. In the presence of NH_4_OH, the solution pH can be adjusted to alkaline conditions, resulting in the reaction products without nitric acid formation. In this respect, reaction (2) can be described as follows:

10 Ca(NO_3_)_2_ + 6(NH_4_)_2_HPO_4_ + 8 NH_4_OH → Ca_10_(PO4)_6_(OH)_2_ + 20 NH_4_NO_3_ + 6 H_2_O (3)

Hydrothermal technique utilizing elevated temperatures (ca. below 350 °C) and pressures in aqueous solutions enables the formation of nHA crystals with homogeneous chemical compositions [87,90,91]. Nagata et al. prepared nHA rods from Ca(NO_3_)_2_ and (NH_4_)_2_HPO_4_ precursors by treating the solutions hydrothermally at 60 °C, 120 °C and 180 °C under a pH of 10 [90]. Xue et al. also obtained HA nanorods by means of hydrothermal route [91]. However, the synthesis of nHA by hydrothermal method is a time consuming and costly process. Instead, microwave (MW) synthesis of nHA offers several advantages including rapid and uniform heating, short synthesis time, narrow particle size distribution, and high purity of the product [92,93,94]. In the MW synthesis, temperature, pH, MW power and duration as well as the use of chelating agent have a pronounced effect on the formed phase structure and morphology [94]. Siddharthan et al. synthesized nHA through a co-precipitation process of calcium nitrate and orthophosphoric acid, followed by microwave irradiation. The morphology of the nHA changed from needle-like to acicular and finally to platelet with the increase in microwave power [92]. Kalita and Verma synthesized highly crystalline nHA using Ca(NO_3_)_2_ and Na_2_HPO4 in the presence of ethylene diamine tetraacetic acid (EDTA) under microwave heating at 600 W; EDTA served as a chelating agent for calcium ions. The as-synthesized nHA possessed rod-shape and elliptical morphologies [93]. 

### 2.2. Graphene Oxide and Its Derivatives

**Figure 4:**
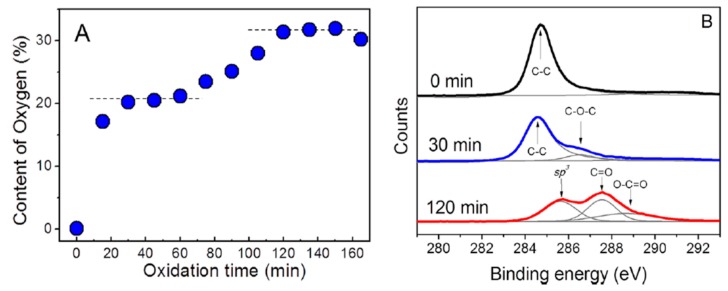
(**A**) Oxygen content in graphene oxide detected by X-ray photoelectron spectroscopy as a function of oxidation time. (**B**) XPS C-1s spectra of GO formed in strong oxidants for 0 min, 30 min (lower plateau in panel A), and 120 min (upper plateau in A) [95]. Reproduced with permission from the American Chemical Society, published by American Chemical Society, 2018.

The synthesis of GO and rGO is well described in the literature, so it is discussed briefly herein [45,95,96]. Modified Hummers process is the most commonly adopted technique for synthesizing GO [97]. In the process, graphite and sodium nitrate are dispersed in sulfuric acid. The round flask with a solution mixture is immersed in an ice bath under continuous stirring. This is followed by slow addition of potassium permanganate. Thereafter, the suspension is diluted with deionized water. The oxidation reaction is terminated by adding a large amount of water and hydrogen peroxide. The reaction mixture is filtered, washed with HCl and deionized water to remove excess of manganese salt. The drawbacks of this process are the generation of toxic gases (NO_2_ and N_2_O_4_) due to the use of sodium nitrate, and the formation of defective GO. In addition, the use of potassium permanganate generates trace amounts of Mn and Fe in GO, which may induce toxicity in mammalian cells [98,99]. From the literature, many researchers tend to use different oxidation times and temperatures, as well as different types and concentrations of oxidants to synthesize GO [100,101,102]. As a result, GOs possess different O contents or C/O ratios, especially those prepared from various oxidation times. Skakalova employed X-ray photoelectron spectroscopy (XPS) to evaluate oxygen content in the GOs prepared from modified Hummers method with different oxidation periods (Figure 4A,B) [95]. During chemical oxidation for the first 60 min, deconvoluted C-1s spectrum shows the main peak at about 284.7 eV and a small shoulder at 286.6 eV, corresponding to the sp^2^ carbon and C–O–C bonded species, respectively. Thus, the first-phase oxygen uptake of ∼20 at% leads to the formation of C–O–C bonded species. The graphitic phase (C–C bond in sp^2^ hybridization) still retains, thus part of the structure is partially intercalated by oxygenated species. The oxygen uptake rises above 30 at% as the oxidation proceeds, and deconvoluted C-1s spectrum shows the presence of sp^3^-hybridized C–C bond species (285.6 eV), C=O (287.6 eV) and O–C=O (288.6 eV) groups, implying a complete oxidative intercalation (Figure 4B). After oxidation for 4 days, the structure of intercalated graphite becomes unstable and spontaneously exfoliates into individual GO sheets. Extended oxidation for two weeks leads to a significant decrease in oxygen content from 30 to 20 at%, due to the spontaneous release of CO_2_ [95]. The different C/O ratios in GOs result in a large variation in their structural properties, as revealed by the differences in the degree of exfoliation, number and thickness of graphene layer, lateral size, functional group content, etc. [97]. 

GO is known to possess oxygen functional groups such as carboxyl, hydroxyl, and epoxide on the basal plane and the edge of graphene sheet [96,103,104]. Thus, GO sheets can be readily hydrated in water because of the presence of carboxyl groups at the edges. GO can also be dispersed in certain organic solvents such as *N*-methyl-2-pyrrolidone (NMP), dimethylformamide (DMF), tetrahydrofuran (THF), and ethylene glycol due to the presence of oxygenated groups [105]. Those groups render GO electrically insulating, thereby restricting its use in the electronic industries. To restore its electrical conductivity, reducing agents, such as hydrazine and sodium borohydride, are employed to reduce GO into rGO [106]. In general, hydrazine is the most commonly used agent because of its good reduction capability for eliminating oxygenated groups. It is noted that chemical reduction of GO to rGO cannot completely eliminate oxygenated groups. The oxygenated groups can also be removed as carbon dioxide by rapid heating GO to 1050 °C under vacuum or an inert atmosphere to generate thermally reduced graphene (TRG) [107]. This high temperature treatment generates structural defects in TRG in the form of etch holes within the graphene basal plane [108]. 

**Figure 5:**
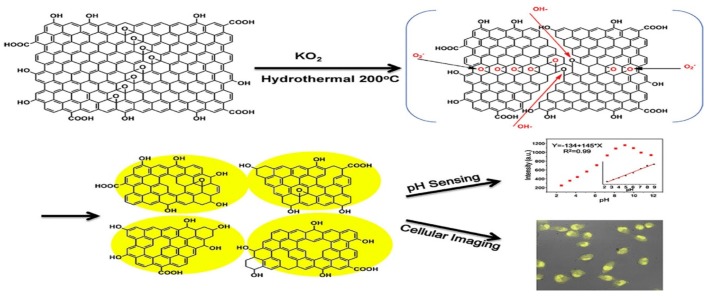
Schematic illustrations of hydrothermal cutting procedure for GO with the assistance of KO_2_ to form graphene quantum dots with yellow photoluminescence [115]. Reproduced with permission from Elsevier, published by Elsevier, 2017.

Graphene quantum dots (GQDs) are 0D carbonaceous nanomaterials having low toxicity, good biocompatibility, tunable photoluminescene, and remarkable quantum confinement effect, rendering them attractive for biomedical applications, e.g., bio-imaging, drug delivery, tissue engineering, etc. [76,79,80,109,110,111,112]. GQD can be prepared from GO by cutting its graphene sheet under hydrothermal treatment, where concentrated H_2_SO_4_ and/or HNO_3_ are employed for oxidizing GO [113,114]. In the process, graphene sheet of GO is cut by mixed sulfuric/nitric acids under ultrasonication. Then the treated suspension is transferred to a heated autoclave to form GQDs [113]. Very recently, Zhao et al. prepared GQDs by dispersing GO and KO_2_ in deionized water under stirring. The suspension was then transferred into an autoclave, and heated at 200 °C for 24 h. With the assistance of KO_2_, a conversion rate of ∼35 wt% from GO to GQDs could be achieved. GQDs displayed yellow emissive photoluminescence that was useful for cellular imaging and pH sensing (Figure 5) [115]. Several chemical preparation methods such as microwave assisted cleaving, solvothermal cutting, and electrochemical method had been used for making GQDs [110,116,117]. For example, Li et al. prepared GQDs by cleaving GO under acidic conditions. The cleaving and reduction processes were accomplished simultaneously using microwave irradiation [116]. Hakkarainen and coworkers employed microwave assisted hydrothermal technique to prepare GQDs from cellulose-rich paper [68]. The paper was soaked in dilute sulfuric acid followed by microwave heating at 180 °C for 2 h.

### 2.3. Graphene Oxide-Nanohydroxyapatite

**Figure 6:**
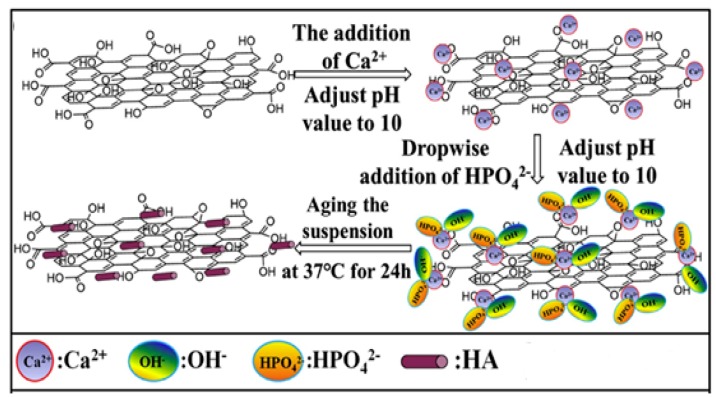
The proposed in situ synthesis mechanism of hydroxyapatite on pristine graphene oxide sheets [127]. Reproduced with permission from the Royal Society of Chemistry, published by the Royal Society of Chemistry, 2013.

**Figure 7:**
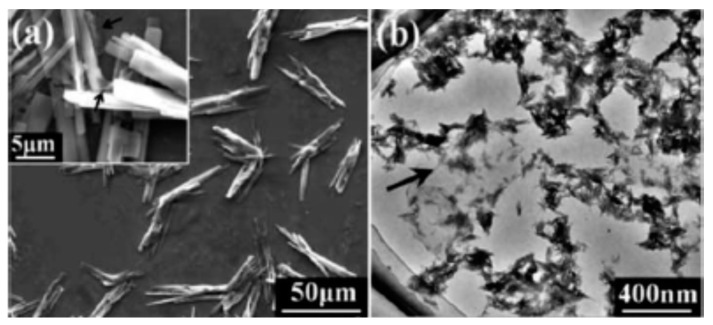
(**a**) Scanning electron microscope and (**b**) transmission electron microscope images of GO/nHA. The inset in (**a**) shows a high magnification image of GO/nHA [127]. Reproduced with permission from the Royal Society of Chemistry, published by the Royal Society of Chemistry, 2013.

**Figure 8:**
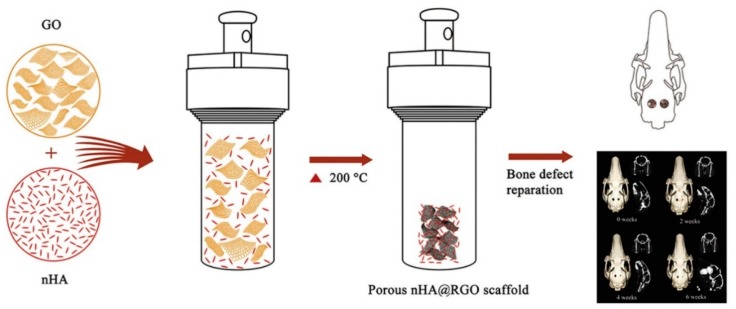
rGO/nHA nanocomposite prepared from self-assembly for bone defect reparation [130]. Reproduced with permission from Elsevier, published by Elsevier, 2017.

GO and nHA can be added simultaneously to biodegradable polymers to form hybrid bionanocomposites [81,118,119,120,121,122,123,124]. GO/nHA hybrids exhibit remarkable improvements in the mechanical performance and osteoconductivity due to the high elastic modulus of GO and excellent biocompatibility of nHA. GO and nHA can be synthesized in situ to generate GO/nHA nanocomposites [125,126,127,128]. Rodríguez-Gonzalez et al. prepared GO/nHA through hydrothermal treatment of an aqueous solution containing GO, Ca(NO_3_)_2_, (NH_4_)_2_HPO_4_ and NH_4_OH at 90 °C [125]. In the process, GO was reduced to rGO under hydrothermal treatment. Li et al. synthesized GO/nHA nanocomposite by adding Ca(NO_3_)_2_ into aqueous GO solution, followed by adjusting the pH of solution to 10 with ammonia water [127]. Then (NH_4_)_2_HPO_4_ solution was dropwise added to the suspension under vigorous stirring, during which the pH was kept at 10 by titration with ammonia water. The reaction product was finally aged at 37 °C for 24 h (Figure 6 and Figure 7). The GO/nHA formation mechanism was ascribed to the electrostatic interaction between the GO and nHA. The negatively charged carboxylic groups at the basal plane edges of GO attract Ca^2+^ cations. Thus, Ca atoms were favorably adsorbed on the edges. Subsequently, HPO_4_^2−^ anions were attracted by Ca^2+^ cations through electrostatic interaction, thereby forming nHA on the plane edges during aging. Analogously, rGO/nHA nanocomposites can also be fabricated in the same way because rGO still contains residual oxygen functional groups [129]. Very recently, Nie et al. prepared rGO/nHA nanocomposite by mixing GO and nHA under sonication to form a homogeneous suspension, followed by hydrothermal treatment at 200 °C to induce self-assembly [130]. Self-assembly is a process in which individual components organize themselves into an ordered structure due to the electrostatic interactions among the components. GO then converts to rGO during thermal treatment (Figure 8). So GO is effectively reduced to rGO without using toxic reducing agent such as hydrazine.

## 3. Preparation of Polymer Bionanocomposite Scaffolds

**Figure 9:**
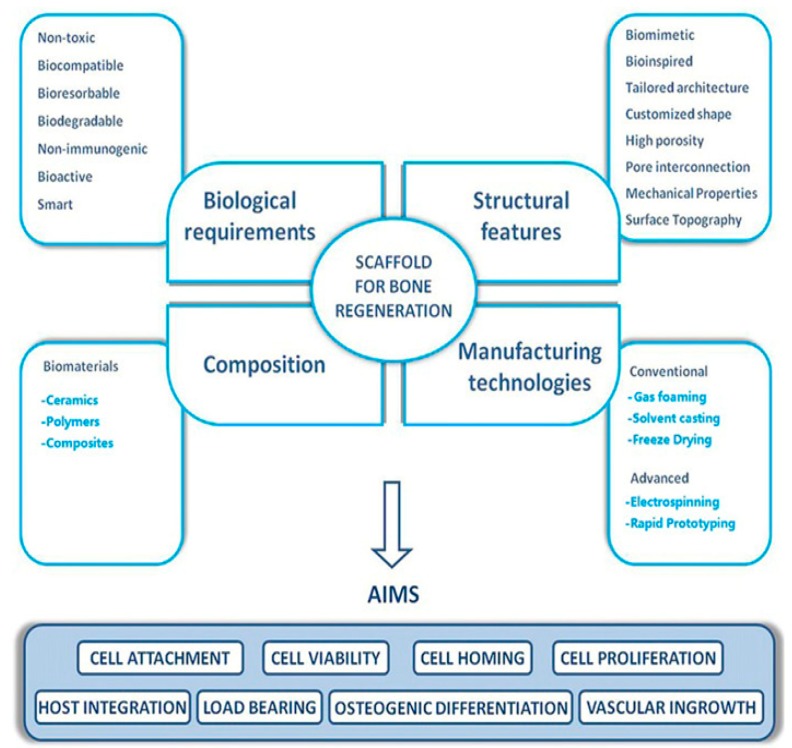
Required properties of an ideal scaffold for bone tissue engineering applications [131]. Reproduced with permission from Elsevier, published by Elsevier, 2017.

Scaffolds for bone tissue engineering must be compatible, biodegradable, bioactive, highly porous with interconnected pore network and good mechanical strength to support bone tissue ingrowth, nutrient transport and metabolic waste removal as mentioned previously (Figure 9) [16,17,131]. Those scaffolds can be prepared from a wide variety of techniques including solvent casting/porogen leaching, gas foaming, freeze drying, thermally induced phase separation, melt- or wet-spinning, and electrospinning [16,132,133]. Each technique has its advantages and limitations. Conventional porous scaffold fabrication such as solvent casting/porogen leaching involves the dissolution of a polymer in an organic solvent, followed by mixing with porogens (e.g., salt, sugar). The polymer solution is then cast into the desired molds, allowing the solvent to evaporate and leaving behind polymer/porogen material. Finally, water is employed to dissolve porogens. The size and pore fractions in the scaffolds are controlled by those of porogens [132]. The major drawbacks of this technique include the use of solvent, long soaking time in water to leach out porogens, and limited membrane thickness (3 mm). Gas foaming can create a porous structure in the presence of pressurized gas such as carbon dioxide, thereby eliminating the use of toxic solvents [133]. This process utilizes supercritical CO_2_ gas acting as a plasticizer and foaming agent to produce porous scaffolds. In foaming process, the polymer is saturated with CO_2_ in the supercritical state, and dense CO_2_ diffuses and plasticizes polymer by reducing its glass transition temperature (Tg). After saturation, CO_2_ is depressurized quickly such that Tg begins to rise and CO_2_ escapes from the polymer matrix owing to a rapid drop in pressure. This leads to the nucleation of bubbles and the formation of foams. The drawbacks of this method are the poor control of pore interconnectivity and the formation of a nonporous skin layer at the scaffold surface [134]. In the freeze-drying technique, a water soluble polymer is frozen such that an interpenetrating ice crystals are created. Those ice crystals are removed by sublimation, resulting in the formation of porous scaffolds [135]. Thermally induced phase separation (TIPS) allows the formation of an interconnected porous structure. It is based on the changes in temperature to induce phase separation of a homogeneous polymer-solvent solution through solid liquid demixing or liquid-liquid phase separation. This causes the formation of a polymer-rich phase and polymer-poor phase upon rapid cooling polymer-solvent solution; the polymer-poor phase is then removed [136,137]. Several parameters such as the types of polymers and solvents, polymer concentrations and molecular weights, cooling temperatures and rates can affect the morphology and the size of pores.

Fibrous scaffolds with a large surface area and a relatively large porosity can be fabricated using melt- or wet-spinning. In melt spinning, a polymer is melted and extruded through a die having many small holes. The molten fibers are then cooled and solidified in air. The resulting fibers are collected by a take-up wheel to form continuous fiber strands. However, the high temperatures employed for melting polymers prevent its use for the encapsulation of bioactive compounds and cells. Wet spinning involves dissolving polymer in an appropriate solvent followed by extruding the polymer solution via a spinneret into a coagulation bath containing a non-solvent. The diameters of wet spun filaments are typically in the range of 30–600 µm. The advantages of wet spinning include intrinsic higher porosity and larger pore size [138,139]. Electrospinning is an effective tool to prepare micro- or nano-fibers using an electric field to manipulate the ejection of polymeric jet from a needle attached to the syringe. In the process, the applied electric field charges the polymer droplet at the needle tip held by its surface tension. At a critical voltage, the droplet elongates into a Taylor cone. By increasing electric field above a critical value, electrostatic repulsion can overcome surface tension of the droplet. So a polymer jet is drawn from the tip of the Taylor cone towards grounded collector. As the jet travels in air, it undergoes whipping motion and solvent evaporation. Electrospun nanofibers with interconnected porous structure and large surface area show morphological similarities to the natural ECM [140,141]. By adding nHA to biodegradable polymers, the resultant fibrous scaffolds favor cell adhesion, growth and differentiation [81,142,143,144]. However, the pores of electrospun scaffolds are rather small, typically about 200–800 nm and below 5 µm, thus restricting bone cell infiltration and vascular ingrowth [145]. In this respect, cell-scaffold interaction is limited to the surface only. As recognized, bone tissues depend greatly on the vascular network to deliver nutrients, oxygen and metabolic waste [146]. Thus, the lack of control of pore size and its associated vascularization limit the widespread use of electrospun mats for clinical applications. 

**Figure 10:**
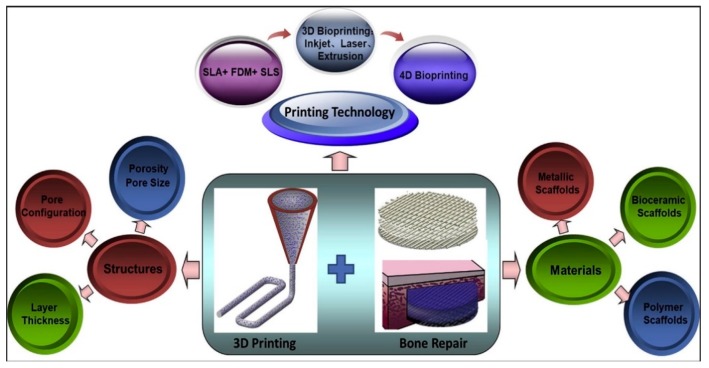
A framework of 3D printing [147]. Reproduced with permission from Elsevier, published by Elsevier, 2019.

**Figure 11:**
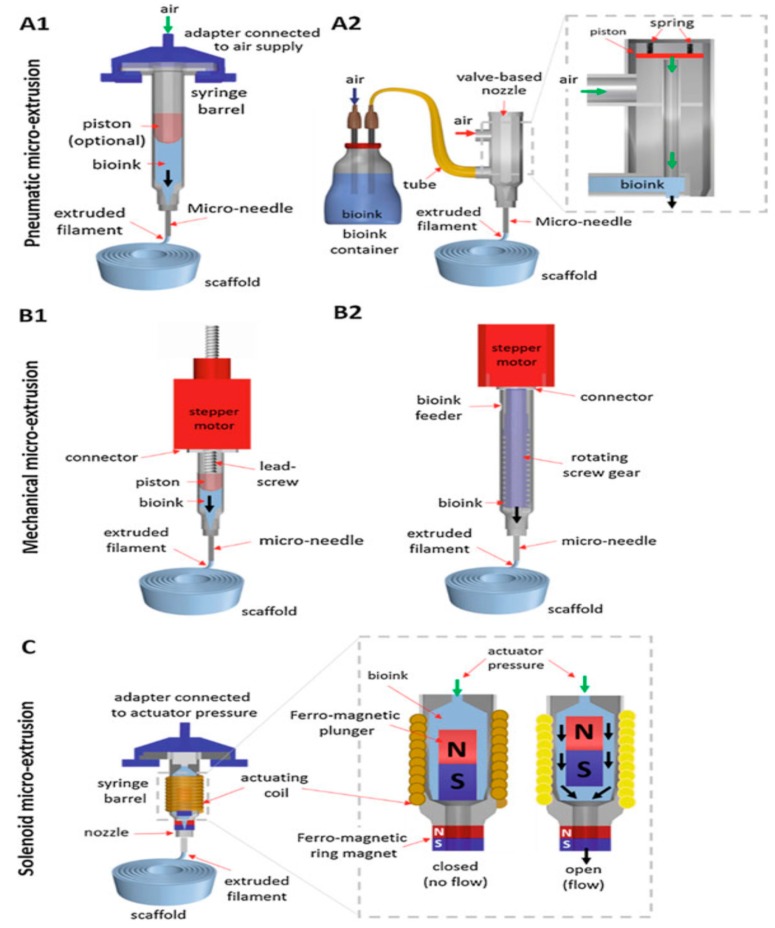
Extrusion-based bioprinting processes: Pneumatic-based extrusion including (**A1**) valve-free and (**A2**) valve-based. Mechanical-based extrusion including (**B1**) piston-based and (**B2**) screw-based, and (**C**) solenoid-based extrusion [151]. Reproduced with permission from Elsevier, published by Elsevier, 2016.

Rapid prototyping (RP), also known as additive manufacturing (AM), has emerged as an effective tool for printing 3D porous scaffolds with well-defined and reproducible architectures using a wide variety of materials (Figure 10). Moreover, this technology can fabricate patient-specific 3D objects with complex geometries. AM scaffolds can be fabricated using different routes, such as stereolithography (SLA), selective laser sintering, fused deposition modeling (FDM), etc. [147,148,149,150,151]. High-resolution SLA employs lithographic method to photocrosslink liquid polymer resins, e.g., acrylics and epoxies by a UV laser. Aliphatic polyesters generally lack biodegradable photoreactive groups for crosslinking [148]. The AM system is typically equipped with a computer aided design (CAD) system for processing a highly porous 3D scaffolds with a controlled architecture in a layer-by-layer mode. The desired implant area of a patient is scanned by the X-ray or computer tomography, and the data are converted into a CAD system. For example, selective laser sintering (SLS) employs a computer-controlled CO_2_ laser beam to selectively fuse and sinter polymer composite powders in a layer-by-layer manner to build up a 3D scaffold. The advantages of SLS for forming polymer scaffolds including material versatility, and capable of producing objects with complicated shapes [150]. However, SLS products with a grainy surface finish suffer from shrinking and warping due to thermal distortion. FDM is the most widely used extrusion-based AM, and offers the advantages of simplicity, flexibility, low cost and ease of fabrication without the use of toxic organic solvents. In FDM, thermoplastic filament is guided into a liquefier for melting through the rollers, followed by extruding in a layer-by layer manner using a computer-controlled nozzle. The quality of 3D printed objects depends greatly on the FDM parameters including the nozzle temperature, nozzle diameter, extrusion speed, layer thickness, raster angle, etc. [147]. FDM suffers from the need for preformed polymer fibers to feed through the rollers and nozzle for melting. Alternatively, AM technique based on extrusion of a polymer solution can be used to fabricate bioactive tissue or cell laden scaffolds. This technique does not require heating process, thus permitting inclusion of cells and bioactive molecules. Several extrusion set-ups including solenoid, pneumatic-, and mechanical- (piston or screw-driven) system have been developed for this purpose (Figure 11) [151]. 

In recent years, 3D ink-printing process is widely used in industrial sector for fabricating optoelectronic and electronic films [43,152]. At present, 3D bioprinting has found potential applications in the fields of tissue engineering, regenerative medicine and pharmaceutics. 3D ink printing involves a layer-by-layer deposition of biomaterials with spatial control of functional components using a liquid ink as shown in Figure 11. For instance, Jakus et al. prepared an ink through simple mixing of graphene suspension and polylactide-*co*-glycolide solution. This ink can be utilized for printing polymer/graphene scaffolds via an extrusion-based 3D printing system [153]. By employing cell encapsulation approach, porous 3D scaffolds are formed by precisely co-printing bioinks of multiple materials consisting of polymers, living cells, genes, growth factors, and extra-cellular matrices. Furthermore, stem cells can adapt readily to tissues, thus they are an attractive option for bioprinting bone tissues [154,155,156,157,158]. The recent progress of 3D bioprinting technology offers a possible way to solve the vascularization issues [157]. However, 3D bioprinting also has certain limitation because it is static and considers only the initial state of the printed object. With the rapid progress of nanotechnology, four-dimensional (4D) bioprinting has been developed to include conformational changes in printed structures very recently. By incorporating time as the fourth dimension and combining 3D bioprinting strategies, the printed objects can change their shapes or functionalities with time under an applied external stimulus (e.g., magnetic field) [159]. 4D printing can be developed also without the use of nanotechnology by exploiting other polymer properties to achieve shape memory [160]. 

### 3.1. PLA-Based Nanocomposites

#### 3.1.1. PLA-nHA Nanocomposites

##### Processing and Properties of PLA/nHA Nanocomposites

Aliphatic polyesters such as PLA, PGA, PLGA and PCL have found useful applications in tissue engineering, such as degradable sutures, bone fixation devices, stent, etc. [28,161,162,163,164,165]. For instance, PLA with a slow degradation rate is typically used for long-term bone plates and screws [28]. However, PGA with a rapid degradation rate is mainly used for sutures and drug delivery carriers [161,164]. Generally, polyesters degrade through hydrolytic chain scission or cleavage of their ester bond linkage, leading to a reduction of their molecular weight. The hydrolysis rates are affected by several factors including temperature, molecular structure, and hydrophilicity/hydrophobicity of polymers. PGA is a semicrystalline polymer, having a high melting temperature ranging from 225–230 °C and a Tg between 35–40 °C. Because of its simple chemical structure and stereo-regularity, its degree of crystallinity varies from fully amorphous to 46–50% [162,164]. PGA is hydrophilic with a low solubility in organic solvents. Thus, it degrades rapidly in an aqueous solution. PLA has a high melting point of 173–178 °C, a Tg of 60–65 °C, an elastic modulus of 2.7–3.8 GPa, and a tensile strength in the range of 48–110 MPa [162,163]. PLA is a chiral polymer having different stereoisomers. These include poly(l-lactide) [PLLA], poly(d-lactide) [PDLA] and poly(dl-lactide) [PDLLA] with different physical, mechanical and biodegradation properties. Isotactic PLLA and PDLA are crystalline, whereas atactic PDLLA is amorphous having no melt point [162]. PLLA is more commonly used for biomedical applications than PDLA. PLLA stereoisomer is referred to as the PLA in this review. PLA is more hydrophobic than PGA due to the presence of a methyl group in its molecular structure. Accordingly, its ester bond is more resistant to hydrolysis, owing to steric hindrance by the methyl group. Thus, PLA degrades at a slower rate than PGA, releasing lactic acid during hydrolysis. To regulate the hydrolysis rate, copolymers of PLA and PGA are synthesized to form PLGA in which the physical properties depend greatly on the ratio of lactic acid (LA) to glycolic acid (GA). PLGA is generally an acronym for poly d,l-lactic-co-glycolic acid where d- and l- lactic acid forms are in equal ratio [164]. A copolymer with a LA to GA ratio of 80:20 is semi-crystalline. When the ratio of monomer LA/GA increases, the degradation rate of the copolymer decreases accordingly. Amorphous copolymer is produced at a ratio of 25 LA:75 GA. PLGA copolymers with different LA/GA ratios are commercially available for biomedical applications in different forms, including mesh, scaffold, hydrogel, and suture [165]. PCL is a semi-crystalline polymer with a low melting temperature (58–63 °C), a very low Tg (−60 °C), and a low elastic modulus of 0.4 GPa [164]. PCL is hydrophobic and degrades much slower than PLA due to its highly crystalline structure. The degradation rates of aliphatic polyesters decreases in the following order: PGA > PDLLA > PLLA > PCL [162]. 

PLA can be produced from renewable agricultural resources, i.e., lactic acid derived from corn starch, and the process involves a microbial fermentation. PLA has been used in clinical sector as the scaffolds and fracture fixation devices, however, several issues may arise from the use of PLA in orthopedics. For instance, hydrolytic release of lactic acid induces local inflammation response, leading to a decline of cell adhesion and cell proliferation [161]. PLA possesses little bioactivity for bio-mineral deposition and osteoconductivity due to its hydrophobic nature. The inherent brittleness and low toughness of PLA limit its usage. Nevertheless, these shortcomings can be overcome by incorporating nHA and GO into PLA to form bionanocomposites. In general, nHA promotes the adhesion and proliferation of osteoblasts on its surface [166,167,168,169], as well as promotes the differentiation of human mesenchymal stem cells (hMSCs) towards osteoblast lineage [170,171,172,173]. Furthermore, addition of alkaline nHA nanoparticles to PLA can neutralize the acidic environment due to the hydrolytic release of lactic acid. Similarly, CVD-graphene sheet and GO also facilitate stem cell growth and differentiation into various lineages [82,83,84,85,174,175,176]. This makes GO an effective filler for bone regeneration. 

Michael et al. injection molded PLA/nHA nanocomposites containing nHA contents from 1 to 5 wt% with or without surface modifying agents [177]. In the absence of surface modifiers, the tensile strength of the PLA/1 wt% nHA nanocomposite is higher than that of neat PLA. Above 1 wt% nHA, the tensile strength of the PLA/nHA nanocomposites reduces considerably due to the agglomeration of nHA and poor interfacial adhesion between nHA and PLA. By treating the surface of nHA with 3-aminopropyl triethoxysilane (APTES), the tensile strength of PLA/nHA nanocomposites improves markedly.

##### Processing and Properties of PLA/nHA Scaffolds

Porous polymeric scaffolds should have sufficient mechanical strength and elastic modulus to support cellular adhesion and growth. Moreover, they should have large pore size and high pore volume for cell infiltration and proliferation. However, polymeric scaffolds with high porosity levels and large pores tend to have poor mechanical strength and stiffness [178]. Therefore, the incorporation of nHA into porous scaffolds can improve their mechanical performance greatly.

**Figure 12:**
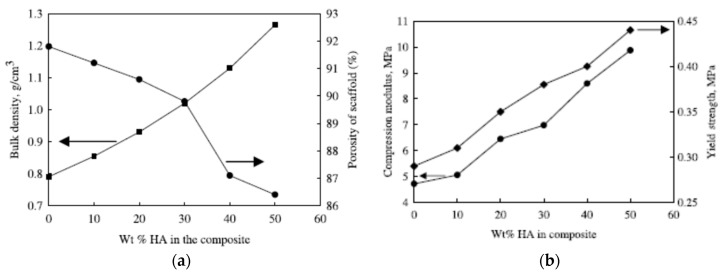
(**a**) Porosity and measured bulk density of the scaffolds vs. nHA content. (**b**) Compressive modulus and strength as a function of nHA content [61]. Reproduced with permission from Elsevier, published by Elsevier, 2005.

**Figure 13:**
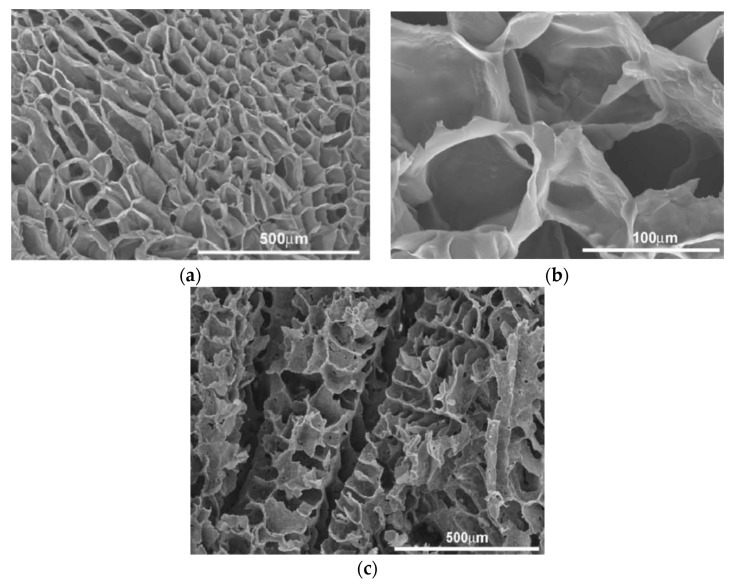
(**a**) Low and (**b**) high magnification SEM images of pure PLA scaffold prepared by thermally induced phase separation process. (**c**) SEM micrograph of PLA/50 wt% nHA scaffold [60]. Reproduced with permission from Elsevier, published by Elsevier, 2004.

Kothapalli et al. fabricated PLA/nHA scaffolds containing 10 to 50 wt% nHA using solvent casting/salt-leaching process [61]. The porosity of PLA scaffold decreased gradually with the increase of nHA content (Figure 12a). The incorporation of nHA into PLA scaffolds increased its compressive modulus and strength markedly. The compressive modulus of PLA scaffold was 4.7 MPa and reached 9.8 MPa at 50 wt% nHA. The compressive strength of PLA scaffold was 0.29 MPa and increased to 0.44 MPa by adding 50 wt% nHA (Figure 12b). The improved mechanical performance of PLA/nHA scaffolds was attributed to a weak ion-dipole interaction between oxygen in the ester group of PLA (C=O) and calcium in nHA. In this respect, applied stress can be transferred effectively from the PLA matrix to nHA fillers during compression testing. Wei and Ma employed TIPS to prepare PLA/nHA scaffolds containing 10–70 wt% nHA via phase separation at −18 °C using dioxane [60]. The morphologies of porous PLA and PLA/50 wt% nHA scaffolds are given in Figure 13a–c respectively, showing a ladder-like pore feature. The pore structure of the scaffolds was created from a phase separation of PLA-dioxant solution. The solvent (dioxant) crystallized during quenching to a cryogenic temperature, and became pores after sublimation [60]. They also reported that the nHA additions improved the compressive modulus of PLA scaffolds. Nejati et al. also prepared porous PLA and PLA/50 wt% nHA scaffolds using TIPS process. A ladder-like pore morphology was also observed in those scaffolds [179]. The mechanical properties of PLA and PLA-nHA scaffolds are listed in Table 1. 

**Figure 14:**
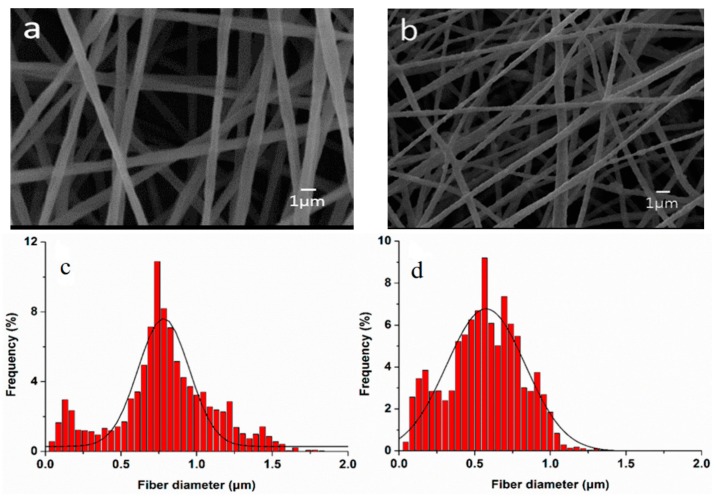
SEM images of electrospun (**a**) PLA, and (**b**) PLA/15 wt% nHA fibrous mates. Fiber diameter distribution of electrospun (**c**) PLA, and (**d**) PLA/15% nHA mats determined by ImageJ software. Reprinted from [81], MDPI under the Creative Commons Attribution (CC-BY) license.

As recognized, ECM consists of fibrous structures made of protein molecules. Much effort has been dedicated by the researchers to fabricate scaffolds that can mimic nanostuctural features of the ECM to promote tissue regeneration. This can be achieved by using electrospinning technique [180,181]. Figure 14a,b shows typical SEM images of electrospun PLA and PLA/15 wt% nHA scaffolds. The inclusion of 15 wt% nHA to PLA reduces its fiber diameters (Figure 14c,d) [81]. Jeong et al. employed electrospinning to fabricate PLA/5 wt% nHA and PLA/20 wt% nHA composite scaffolds [182]. They reported that the nHA additions increase Young’s modulus, tensile strength and tensile ductility of PLA significantly. So nHA with an elastic modulus of 110 GPa can stiffen PLA having low modulus of 2.7–3.8 GPa [70,162,163,164]. The tensile elongation of PLA increases from 27% to 30% and 36%, by adding 5 wt% and 20 wt% nHA, respectively (Table 1). 

As mentioned above, electrospun scaffolds with small pore sizes hinder cell infiltration and ingrowth of bone tissue [145]. Generally, large macropores (200–400 μm) promote the migration of osteoblasts and osteoprogenitors into the scaffolds, and facilitate tissue formation and mineralization [183,184]. However, porous scaffolds with large pores, high porosity level and interconnected pores cannot be well-controlled using conventional fabrication techniques. Accordingly, 3D printing has emerged as an attractive approach to form 3D scaffolds with controlled porosity and large pore size [147,185,186]. In particular, FGM is the most common technique for printing neat PLA scaffolds [187,188,189,190,191,192]. The resulting scaffold had a pore size of 500 μm and 60% porosity. Huang et al. employed low-temperature additive manufacturing (LAM) technology to print PLA/nHA scaffolds with different nHA contents, i.e., 10, 20, 30 and 40 wt% nHA at −20 °C [190]. PLA generally lacks thermal stability at high processing temperature, so low temperature printing can avoid fast degradation of PLA. The pore parameters and mechanical properties of these scaffolds are tabulated in Table 1. PLA/20 wt% nHA scaffold exhibits the highest porosity level of 85.1% and largest pore size of 392 µm. The pore size of this scaffold is large enough for osteoblastic cell adhesion, infiltration and proliferation. Corcione et al. first prepared PLA/nHA filaments followed by printing 3D scaffolds using FDM [191]. From the SEM morphology and compression test results, nHA was found to be uniformly dispersed within the PLA matrix of PLA/nHA scaffolds, thereby improving the mechanical properties of PLA. 

#### 3.1.2. PLA/GO Nanocomposites

##### Processing and Properties of PLA/GO Nanocomposites

Graphene oxide with higher elastic modulus is more effective than nHA to stiffen PLA. GO with large surface area and oxygenated functional groups interacts with PLA through hydrogen bonding, resulting in its uniform dispersion in the PLA matrix, thereby forming PLA/GO nanocomposites with improved mechanical strength at low filler contents. Pinto et al. reported that the addition of 0.4 wt% GO to PLA increases its elastic modulus and tensile strength significantly [193]. Arriagada et al. reported that GOs are more effective than TRGs in enhancing tensile modulus of PLA. The oxygenated groups of GOs enable homogeneous dispersion of nanofillers in the PLA matrix [194]. In general, a strong filler-polymer bonding facilitates an efficient stress-transfer from the polymer matrix to fillers during mechanical testing. As such, the fillers can bear applied stress effectively, leading to a remarkable improvement in the mechanical strength of polymer nanocomposites. A strong filler-matrix bonding can be further achieved in the PLA/GO nanocomposites either by adding a compatibilizer [195], or by grafting GO surface with the PLA or PEG groups [196]. In the latter case, Li et al. grafted GO with PLA to generate GO-*g*-PLA, and then mixed with PLA to form PLA/GO-*g*-PLA nanocomposites using solvent casting process [196]. By adding 0.5 wt% GO-*g*-PLA, the tensile strength of PLA increases from 35 MPa to 72 MPa (an enhancement of 105.7%), while the elongation at break of PLA increases from 6.50 to 14.48% (an improvement of 122.8%).

**Figure 15:**
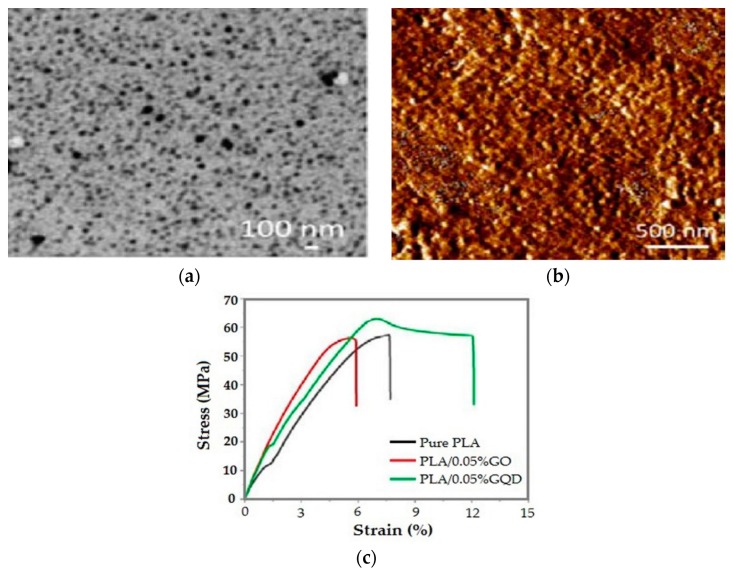
(**a**) TEM image of graphene quantum dots (GQDs). (**b**) Atomic force microscope (AFM) image of PLA/GQDs showing homogeneous dispersion of GQDs in the PLA matrix. (**c**) Stress–strain curves of PLA, PLA/0.05 wt% GO and PLA/0.05 wt% GQD [72]. Reproduced with permission from the American Chemical Society, published by the American Chemical Society, 2016.

More recently, Hakkarainen and coworkers fabricated dense PLA/GQD nanocomposite through the addition of 0.05 wt% GQD (46 nm) using solution coagulation method (Figure 15a,b) [72]. They reported that GQDs were uniformly dispersed in the PLA matrix due to the point-point contact created between adjacent GQDs as a result of their small size, and to the interaction between oxygenated groups of GQDs and PLA. Consequently, the tensile strength and tensile elongation of PLA/0.05 wt% GQD nanocomposite were much higher than those of PLA and PLA/0.05 wt% GO (Figure 15c). Moreover, the GQDs with higher hydrophilicity accelerated the degradation of PLA greatly. 

**Figure 16:**
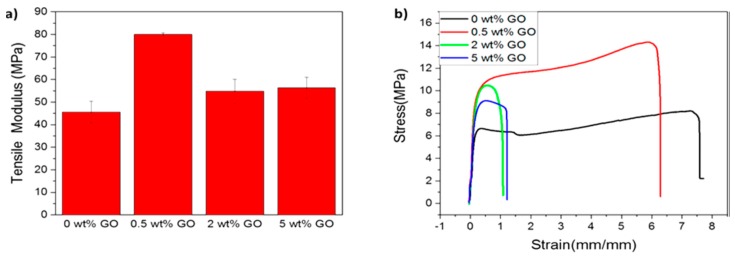
(**a**) Tensile modulus of fused deposition modeling-printed thermoplastic polyurethane (TPU)/PLA blend and its nanocomposites with different GO loadings. (**b**) Tensile stress–strain curves of printed TPU/PLA blend and its nanocomposites with different GO loadings [198]. Reproduced with permission from the American Chemical Society, published by the American Chemical Society, 2017.

FDM technology has been increasing used to print polymer tensile specimens for evaluating their mechanical performance in recent years [197]. To achieve high flexibility in the printed products, Chen et al. combined thermoplastic polyurethane (TPU), PLA and GO (0.5, 2 and 5 wt%) to print solid FDM specimens with high strength and flexibility [198]. TPU is an elastomer with highly flexible and transparent properties, and compatible with living cells. Therefore, the combination of PLA and TPU in different concentration ratios can lead to the formation of ductile and bendable polymer blends that are suitable for tissue engineering applications. In the printing process, nanocomposite filaments are first produced by mixing all constituent materials in organic solvents followed by solvent evaporation, drying and extrusion. The as-produced filaments are then guided by the rollers into a FDM printer to fabricate tensile bars. Chen et al. demonstrated that the addition of 0.5 wt% GO to TPU/PLA 70/30 blend largely improves the tensile modulus and strength as well as thermal stability of the nanocomposite. The average tensile modulus and yield strength of FDM-printed TPU/PLA 70/30 are 45.56 MPa and 6.65 MPa, respectively. By adding 0.5 wt% GO, the average tensile modulus and yield strength increase to 79.96 MPa and 11.25 MPa, respectively. The average strain at break of TPU/PLA 70/30 is 717%, and reduces to 602% by adding 0.5 wt% GO (Figure 16).

##### Processing and Properties of PLA/GO Scaffolds

**Figure 17:**
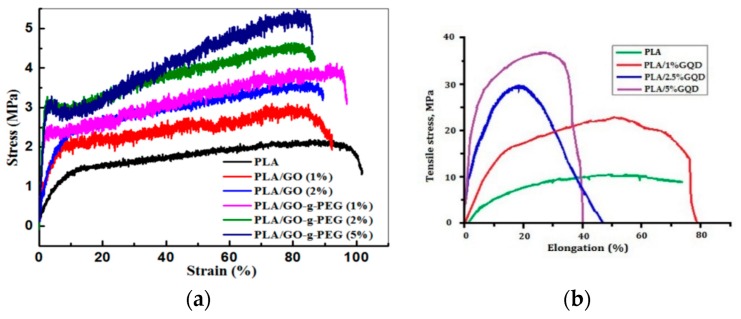
(**a**) Tensile stress–strain curves of electrospun PLA, PLA/GO and PLA/GO-*g*-PEG scaffolds. Reproduced from [200] with permission of Elsevier. (**b**) Tensile stress–strain curves of electrospun PLA and PLA/GQD scaffolds. Reproduced from [76], MDPI under the Creative Commons Attribution (CC BY) license (http://creativecommons.org/licenses/by/4.0/).

Very recently, Mao et al. prepared electrospun PLA/GO mats with different GO concentrations [199]. The GO additions markedly improve the mechanical properties and thermal stabilities of nanofibrous mats. Zhang et al. surface grafted GO with PEG to enhance its interfacial bonding with PLA. They then introduced GO and GO-*g*-PEG nanofillers into PLA to form electrospun fibrous scaffolds [200]. The diameter of PLA decreases from 839 nm to 706 nm by adding 1 wt% GO, but increases to 863 nm due to the 2 wt% GO addition. Comparing with PLA scaffolds, the fibers of PLA/GO-*g*-PEG scaffolds have finer diameters. Generally, two competing processing factors can affect the diameters of electrospun composite scaffold, i.e., viscosity and electrical conductivity of the polymer solution. High solution conductivity tends to produce fine fibers, while large solution viscosity leads to the formation of coarse fibers. The electrical conductivity of PLA solution is 1.19 µS·cm^−1^. The conductivity increases to 2.08 µS·cm^−1^ and 2.70 µS·cm^−1^ by adding 1 wt% GO and 2 wt% GO, respectively [200]. The GO solid film is generally known to be an electrical insulator. However, its oxygenated groups renders it with a negative surface charge in the solution. The solution conductivity depends on the presence of ions in the solution. As such, the conductivity of PLA/GO solutions increase with increasing GO content. The viscosity of the PLA/GO solutions also increases with increasing filler content as expected. Thus, the conductivity effect is the dominating factor in reducing the average diameter of PLA/1 wt% GO. Meanwhile, the increase in the mean diameter of PLA/2% GO can be attributed to a large increase in the solution viscosity as a result of 2 wt% GO addition. Figure 17a shows the tensile stress–strain curves of electrospun PLA/GO and PLA/GO-*g*-PEG scaffolds. Apparently, GO-*g*-PEG nanofillers are more effective than GO in improving the tensile strength of PLA at the same filler loading. The grafted PEG chains of GO can enhance filler dispersion in the PLA matrix and interfacial interaction between the GO and PLA. The tensile strength of PLA is 2.1 MPa, and increases to 2.9 MPa with 2 wt% GO, while to 4.5 MPa at 2 wt% GO-*g*-PEG. It is noted that both nanofillers only led to a slight decrease in tensile elongation of composite scaffolds. The fiber diameters and tensile properties of PLA/GO and PLA/GO-g-PEG scaffolds are listed in Table 1. We now consider the effect of GQD additions of the tensile properties of PLA. Hakkarainen and coworkers introduced 1–5 wt% GQDs into PLA scaffolds during electrospinning [76]. Comparing with PLA, a remarkable enhancement of elastic modulus and tensile strength can be achieved by adding GQDs. These tensile parameters increase with increasing GQD content (Figure 17b). 

#### 3.1.3. PLA/nHA-GO Hybrid Nanocomposites

##### Processing and Properties of PLA/nHA-GO Hybrid Nanocomposites

**Figure 18:**
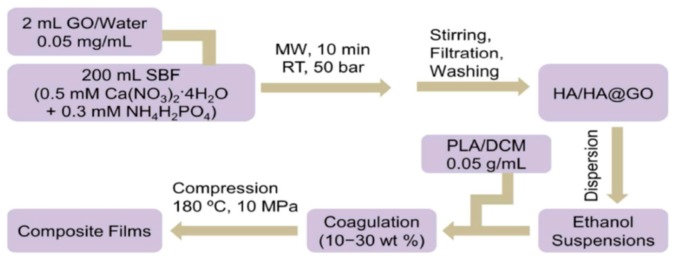
Schematic showing synthetic strategies for nHA and nHA-GO under microwave-assisted heating. The solution coagulation and compression molding were used to fabricate dense PLA/nHA and PLA/nHA-GO films [201]. Reproduced with permission from the American Chemical Society, published by the American Chemical Society, 2018.

**Figure 19:**
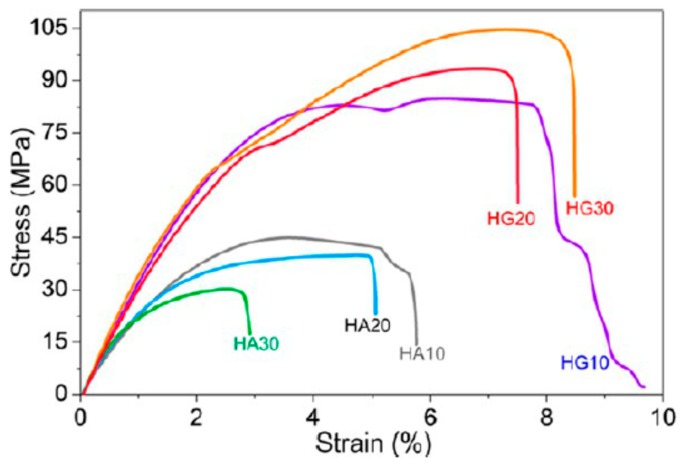
Stress–strain curves of dense PLA/nHA-GO nanocomposite films showing enhanced tensile strength and ductility. PLA/nHA nanocomposite films with 10, 20 and 30 wt% nHA were denoted as HA10, HA20, and HA30, respectively. PLA/nHA-GO hybrid films with 10, 20 and 30 wt% nHA-GO were denoted as HG10, HG20, and HG30, respectively [201]. Reproduced with permission from the American Chemical Society, published by the American Chemical Society, 2018.

Very recently, Chen et al. fabricated dense PLA/nHA-GO hybrid nanocomposite films using coagulation process [201]. In the process, calcium nitrate and ammonium dihydrogen phosphate were dispersed in a simulated body fluid (SBF) solution followed by adding aqueous GO solution. Microwave irradiation was employed to heat the mixed solution to generate nHA-GO with 1 wt% GO. Pure nHA was also prepared using the same processing conditions (Figure 18). Compact PLA/nHA-GO films with 10 to 30 wt% filler contents were fabricated by slowly dropped ethanol suspension of nHA-GO into PLA/dichloromethane (DCM) followed by coagulation. The coagulated products were finally compression molded into dense thin films. PLA/(10–30 wt%) nHA films without GO were also prepared for comparison purposes. Tensile test results showed that nHA-GO fillers were more effective than nHA in improving the tensile modulus, tensile strength and elongation at break of PLA (Figure 19). The tensile modulus, tensile strength and elongation of compact PLA film were 1303 ± 108 MPa, 58.6 ± 2.7 and 6.2 ± 0.4%, respectively. By adding 20 wt% nHA, the values of those tensile parameters became 2293 ± 142 MPa, 39.6 ± 3.9 MPa and 5.0 ± 0.4%, respectively. The addition of 20 wt% nHA led to enhanced tensile modulus and strength at the expense of tensile ductility. By incorporating 20 wt% nHA-GO hybrid to PLA, further enhancement of tensile properties was obtained. The esterification between carboxyl group of GO and hydroxyl group of nHA led to a significant improvement in the tensile properties of PLA/nHA-GO films. The tensile modulus, strength and elongation of break of the PLA/20 wt% nHA-GO film were reported to be 3513 ± 182 MPa, 93.2 ± 4.9 MPa and 7.4 ± 0.8%, respectively. The tensile properties of the as-produced PLA and its nanocomposites are better than those of human cancellous bone having an elastic modulus and tensile strength of 67 MPa and 54 MPa, respectively [202]. Thus, these materials with comparable tensile strength to cancellous bone can be used as load-bearing fixation devices for bone repair treatment. 

##### Processing and Properties of Hybrid PLA/nHA-GO Scaffolds

Porous scaffolds made from a single material are generally difficult to fulfill the requirements of tissue engineering because of their intrinsic material property limitations, e.g., the brittleness of ceramic fillers, poor osteoinduction of aliphatic polyesters due to the release of acidic degradation products. In this context, combining two or more types of nanofillers is very effective to make bone tissue scaffolds with improved mechanical performance and good biocompatibility. Recently, a variety of polymeric bionanocomposites can be created by adding two nanofillers with significantly different physical and chemical properties to a single polymer matrix [51,52,53,54,55,81,118,123,203,204,205]. Accordingly, novel hybrid polymer nanocomposites with tailored properties can be developed by bringing the combined advantages of different nanofillers. By inheriting good biocompatibility of nHA and high stiffness of GO, the resultant PLA/nHA-GO nanocomposites are expected to possess excellent bioactivity, osteoconductivity and high mechanical strength.

Tjong and coworkers prepared electrospun PLA/15 wt% nHA-(1–3 wt%) GO scaffolds [81]. The results revealed that hybrid addition of 1 wt% GO or 2 wt% GO to the PLA/15 wt% nHA increases its tensile modulus and strength, especially a marked increase in the elastic modulus. At 3 wt% GO, both the tensile modulus and strength of PLA/15 wt% nHA-3% GO decreased markedly due to the agglomeration of nanofillers (Table 1). 

### 3.2. PLGA-Based Nanocomposite Scaffolds

Poly(glycolic acid) exhibits fast degradation rate in physiological solutions, thus hindering its clinical applications. In this context, PGA is always copolymerized with PLA in different lactide/glycolide concentrations to yield PLGA with intermediate degradation rates between the PLA and PGA. Conventional solvent casting/particulate leaching (SC/PL) involves the use of organic solvents that are harmful to biological cells and tissues. To solve this issue, Kim et al. combined gas foaming and particulate leaching (GF/PL) to prepare PLGA/nHA scaffolds without using organic solvents [206]. The GF/PL scaffolds exhibited interconnected porous structures and superior mechanical performance compared with those of scaffolds prepared by the SC/PL method. Jose et al. prepared electrospun PLGA nanocomposite scaffolds with 1, 5, 10 and 20 wt% nHA [207]. They reported that the storage modulus (determined from the dynamic mechanical analysis) of the scaffolds increased from 441 MPa for pure PLGA to 724 MPa for 5 wt% nHA. Further increase in filler content causes a reduction in the storage modulus, e.g., 371 MPa for 20 wt% nHA, due to the filler agglomeration at high filler loading. It is generally known that the properties of electrospinning solutions including polymer concentration, solution viscosity, solvent, electrical conductivity, surface tension and polymer molecular weight, and processing parameters such as electrical voltage, spinning temperature, flow rate, spinning speed, needle tip to collector distance, etc., affect the morphologies of electrospun fibers greatly [208,209]. Luo et al. prepared a suspension of 15 wt% PLGA or 18 wt% PLGA with 1% GO by dispersing them in a mixed solvent consisting of tetrahydrofuran (THF) and DMF [78]. The PLGA/GO suspension was then electrospun to produce fibrous mats. They reported that the water contact angles of 15%-PLGA and 18%-PLGA nanofibrous mats decreased from 123 ± 4° to 115 ± 4°, and from 122 ± 4° to 111 ± 4°, respectively by adding 1 wt% GO. Thus, GO slightly reduced hydrophobic behavior of PLGA.

Very recently, Fu et al. demonstrated that the additions of 10 wt% nHA, 2 wt% GO, and 10 wt% nHA + 2 wt% GO to fibrous PLGA (75/25) scaffold can reduce its water contact angle <90°, especially for the GO addition [123]. The water contact angle of nanofibrous PLGA mat was 104.1 ± 5.2°, and decreased to 95.2 ± 1.9° and 86.9 ± 6.9° by adding 10 wt% nHA or 2 wt% GO, respectively. Moreover, the contact angle further reduced to 74.4 ± 3.5° for the PLGA/10 wt% nHA-2 wt% GO hybrid scaffold. The beneficial effect of GO in reducing the contact angle of hydrophobic PLGA was pronounced. This effect arouse from the presence of hydrophilic OH, C–O–C and COOH groups on the GO surface [123]. Considering the mechanical properties, the incorporation of 10 wt% nHA into PLGA increased its tensile strength from 2.72 MPa to 3.02 MPa. By adding 2 wt% GO, the tensile strength of PLGA further increased to 5.98 MPa. A maximum tensile strength was achieved by incorporating 10 wt% nHA + 2% GO hybrid nanofillers into the PLGA matrix. 

### 3.3. PCL-Based Nanocomposites 

#### 3.3.1. PCL/nHA Nanocomposites 

##### Properties of PCL/nHA Nanocomposites

PCL, with a high degree of crystallinity and poor hydrophilicity, exhibits very slow degradation rate, thus restricting its use for bone tissue engineering applications. Its degradation rate can be modified by adding hydrophilic nHA, GO, or hybrid nHA/GO nanofillers. Moeini et al. prepared in situ PCL/20 wt% nHA nanocomposite by dissolving PCL in acetone under stirring, followed by adding Ca(OH)_2_ and H_3_PO_4_ to the polymeric solution_._ The mixed solution was treated under solvothermal conditions [210]. The PCL/HA nanocomposite was cast onto a Petri dish and dried for 24 h at room temperature. The tensile modulus and tensile strength of cast PCL film are 106 ± 6 MPa and 9.3 ± 0.3, respectively. The tensile modulus of cast PCL film is somewhat smaller than that of melt-compounded PCL (i.e., 337 MPa) [211]. This may due to the formation of few pores inside cast PCL film as a result of solvent evaporation. By adding 20 wt% nHA to PCL, the tensile modulus and tensile strength increase to 126 ± 4 MPa and 11.8 ± 0.2 MPa, respectively. In addition, the compressive modulus and compressive strength of PCL film are 160.8 ± 7 MPa and 15 ± 0.2 MPa, while those of the PCL/20 wt% nHA are 241.6 ± 12 MPa and 19 ± 0.4 MPa, respectively. Apparently, the addition of 20 wt% nHA to PCL improves its mechanical performance. Eshraghi and Das employed SLS to fabricate bulk PCL film [212]. The tensile modulus of bulk PCL ranged from 343.9 to 364.3 MPa, while its tensile strength ranged from 10.5 to 16.1 MPa. Moreover, the compressive modulus of bulk PCL ranged from 297.8 to 317.1 MPa, and the compressive strength was 38.7 MPa. 

##### Structure and Properties of Porous PCL/nHA Scaffolds

Moeini et al. also prepared porous PCL/20 wt% nHA nanocomposite scaffold with porosity >90% by means of solvent casting/particulate leaching method [210]. The presence of pores with sizes in the range of 10–200 µm in the scaffold led to a drastic reduction in the compressive modulus from 241.6 ± 12 MPa to 1.99 ± 0.076 MPa, and the compressive strength from 19 ± 0.4 MPa to 159 ± 7 kPa. Very recently, Li et al. fabricated electrospun PCL/nHA mats with high nHA loadings of 30 wt% and 60 wt% to promote bone cell infiltration [213]. The water contact angle of PCL was 135.4°, and decreased to 134.1° and 131.8° by adding 30% nHA and 60% nHA, respectively. The pore size of electrospun PCL mat was 1.94 µm, and slightly increased to 2.19 µm and 2.30 µm by adding 30% nHA and 60% nHA, respectively. Li et el. demonstrated that the nHA additions increase the tensile strength and tensile elongation of PCL significa ntly. The PCL mat had a tensile strength of 12.3 ± 0.89 MPa, and increased to 85.17 ± 2.61 MPa and 158.1±12.6 MPa respectively by incorporating 30% and 60% nHA into PCL. Moreover, the tensile elongation of PCL, PCL/30% nHA and PCL/60% nHA were 380%, 530% and 564%, respectively. From these data, it is apparent that the pore sizes of electrospun PCL and its nanocomposite scaffolds are very small for bone cell infiltration. 

AM technique is an effective tool for fabricating 3D PCL and PCL/nHA scaffolds with well-defined macropores for cell infiltration [214,215,216]. SLS is typically used to fabricate PCL scaffolds due to its good processability as a result of low melting (58–63 °C) and glass-transition temperatures (–60 °C) [162,164]. The compressive mechanical properties of SLS-fabricated scaffolds depend largely on the scaffold design. Williams et al. demonstrated that SLS-prepared PCL scaffolds can be easily adapted to fit complex anatomic locations. Compressive modulus and yield strength values of 3D PCL scaffolds range from 52 to 67 MPa, and 2.0 to 3.2 MPa for a design porosity >63%, respectively [214]. Xia et al. employed SLS technique to fabricate cylinder-shaped PCL/(5–15%) nHA scaffolds having pore sizes ranged from 600 to 800 µm [215]. The nHA additions were beneficial to enhance hydrophilicity of PCL. The water contact angle of PCL was 112.98°, and reduced drastically to 87.42°, 81.00° and 79.50° by adding 5 wt%, 10 wt% and 15 wt% nHA, respectively to PCL. So large pore sizes and hydrophilic behavior of PCL/nHA scaffolds favored the adhesion, proliferation and infiltration of hMSCs. Furthermore, nHA fillers were also very effective in improving the compressive strength of SLS-processed PCL. The compressive strength of PCL was 1.38 ± 0.16 MPa, and increased to 2.67 ± 0.20 MPa and 3.17 ± 0.11 MPa by adding 10 wt% nHA and 15 wt% nHA, respectively. 

From the literature, an extrusion-based printing system have been used to print 3D PCL, and PCL/nHA scaffolds [216,217]. Huang et al. employed both nHA and β-tricalcium phosphate (TCP) fillers (10 and 20 wt%) to reinforce 3D PCL/nHA and PCL/TCP nanocomposite scaffolds. As recognized, beta-TCP material finds attractive application as a bone substitute material in orthopedics due to its good biocompatibility, and biodegradation. Their results showed that all scaffolds exhibited a well-defined pore architecture with a uniform pore distribution. The pore sizes of the PCL, PCL/nHA and PCL/TCP scaffolds were in the range of 287 and 317 µm [217]. Furthermore, both nHA and TCP fillers increased the compressive strength of PCL scaffolds. The additions of 20 wt% nHA and 20 wt% TCP increased the compressive modulus of PCL scaffolds from 48.08 ± 0.09 MPa to 75.72 ± 0.57 MPa and 88.07 ± 1.91 MPa, respectively. It is evident that the compressive modulus of 3D printed PCL/20 wt% nHA scaffold is significantly higher than that of solvent-cast PCL/20 wt% nHA scaffold (1.99 ± 0.076 MPa) [210]. Moreover, SLS-processed PCL/15 wt% nHA exhibits considerably higher compressive strength (3.17 ± 0.11 MPa) than that of solvent-cast PCL/20 wt% nHA scaffold (159 ± 7 kPa) [210,215]. Comparing to conventional scaffold fabrication processes, it appears that 3D AM techniques can produce mechanically strong and stable scaffolds with a well-defined porous structure for bone tissue engineering applications. 

**Figure 20:**
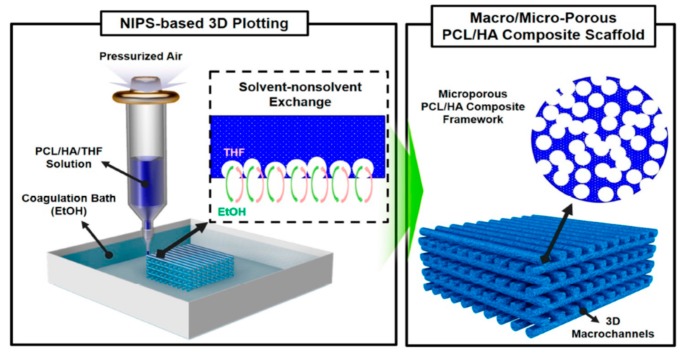
Experimental set-up for non-solvent-induced phase separation (NIPS)-based 3D printing of scaffolds with macro- and micropores. Reproduced from [221], MDPI under the Creative Commons Attribution (CC BY) license (http://creativecommons.org/licenses/by/4.0/).

**Figure 21:**
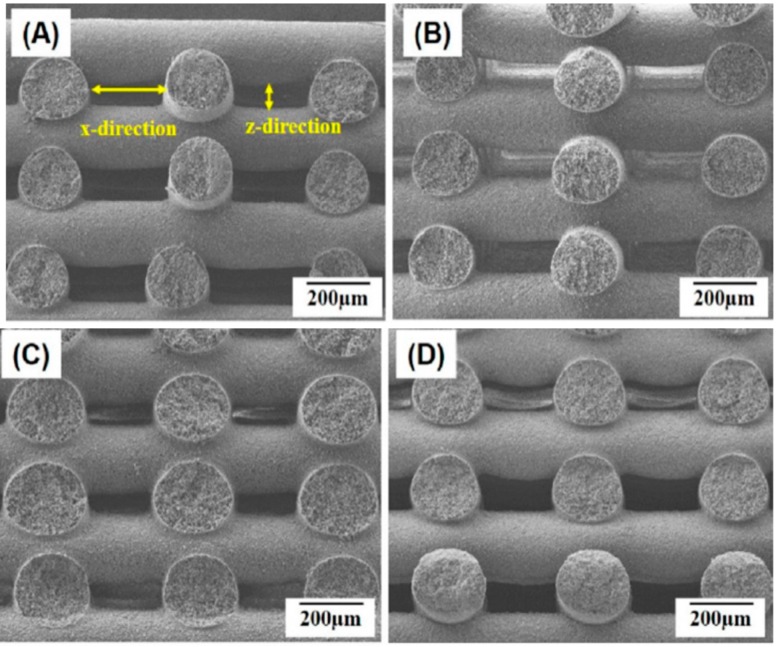
SEM cross-sectional images of NIPS-based 3D printed PCL/nHA scaffolds with different nHA loadings: (**A**) 0 wt%, (**B**) 10 wt%, (**C**) 15 wt% and (**D**) 20 wt%. Reproduced from [221], MDPI under the Creative Commons Attribution (CC BY) license (http://creativecommons.org/licenses/by/4.0/).

**Figure 22:**
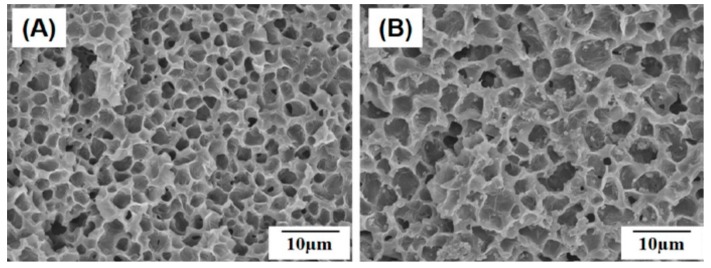
SEM images of micropores formed in the filaments of NIPS-based 3D printed (**A**) PCL and (**B**) PCL/10 wt% nHA scaffolds. Reproduced from [221], MDPI under the Creative Commons Attribution (CC BY) license (http://creativecommons.org/licenses/by/4.0/).

Chiellini and coworkers employed AM technique to fabricate scaffolds with dual-scale porosity at the micro- and macro-levels based on wet-spinning of PCL, PCL/nHA, star-PCL and star-PCL/nHA solutions [218,219,220]. Using the same approach, Kim et al. constructed 3D PCL and PCL/(10–20 wt%) nHA scaffolds with macro-and micro-porous structures through non-solvent-induced phase separation (NIPS)-based 3D plotting. This technique creates macropores between the scaffold filaments based on the printed design, and micropores in the filaments through the phase separation in PCL/HA solutions under non-solvent/solvent exchange mechanism [221]. In the process, nHA is dispersed in the PCL/THF, and the suspension is extruded into an ethanol (EtOH) bath by a nozzle. The filament was deposited in a layer-by-layer sequence of 0°/90° pattern for obtaining macropores with a square geometry (Figure 20 and Figure 21). Micropores in the filaments are created by means of phase separation via the exchange of the solvent (THF) and non-solvent (EtOH). Micropores in the filaments mimic the structure of human bones, thereby providing effective sites for the cell adhesion and nutrient transport [222]. Therefore, the formation of a hierarchical structure in the printed 3D scaffolds consisting of macropores and micropores facilitates osteoconduction and osteointegration in vivo greatly [223,224]. The filament width and porosity of PCL are 219 ± 16 µm, and 78.4 ± 1.2 µm, respectively, while those of PCL/10 wt% nHA are 270 ± 3 µm and 77.04 ± 3.5 µm, respectively. Figure 22A,B are representative SEM images showing uniformly distributed of micropores within PCL filaments or PCL/nHA filaments.

It is noted that the specific pore shapes of 3D printed scaffolds would affect their mechanical and biological properties [225,226]. In other words, the architecture or lay-down pattern of the filaments influences the mechanical behavior and cell adhesion of printed scaffolds. A PCL scaffold with a simple 0/90° pattern generally exhibits square interconnected pores as shown in Figure 21a. By varying the layer deposition angle to 0/60/120° and 0/45/90/135°, scaffolds with triangular and complex internal pore geometries are produced [225]. The printed 3D scaffolds with the orthogonal layer design of 0/90° orientation generally gives better mechanical performance compared to other deposition layer architecture. The scaffolds with a 0/90° pattern exhibit higher compressive modulus and compressive strength than those obtained for 0/60/120° and 0/45/90/135° patterns. The compressive modulus of PCL scaffolds with 0/90°, 0/60/120° and 0/45/90/135° patterns at a filament distance (center to center of filaments) of 650 μm are 34.2 ± 3.8 MPa, 30.5 ± 4.5 MPa and 19.1 ± 2.8 MPa, respectively [225]. 

#### 3.3.2. PCL/GO Nanocomposites

##### Properties of PCL/GO Nanocomposites

In recent years, GO has emerged as an important group of filler material for polymers to form structural and functional polymer nanocomposites due to the notable improvement in their mechanical and electrical properties at low filler loadings. The incorporation of GO into a low modulus polymer matrix can lead to a significant reinforcement [227]. Wan and Chen prepared solid PCL/GO films with different GO loadings of 0.3–2 wt% by means of solvent casting followed by compression molding to obtain dense solid films [228]. The found that GO nanofillers stiffen and reinforce PCL effectively. By adding 0.3 wt% GO, the elastic modulus, tensile strength and tensile elongation of PCL increased from 209 ± 21 MPa, 14.2 ± 1.6 MPa and 554 ± 72%, to 230 ± 30 MPa, 25.1 ± 4.1 MPa and 802 ± 65%, respectively. Comparing with pure PCL control, the elastic modulus and tensile strength of PCL were increased by 10% and 77%, respectively due to the 0.3 wt% GO addition. Increasing GO content to 2 wt%, the elastic modulus and tensile strength further increased to maximum values of 442 ± 35 MPa and 27.5 ± 5.7 MPa, respectively, but tensile elongation reduced to 548 ± 81%. The reinforcing effect of GO on PCL was attributed to the formation of hydrogen bonding between the GO and PCL as verified by Fourier transform infrared spectroscopy (FTIR), and to the homogeneous dispersion of GO in the PCL matrix. 

As mentioned, GO is an electrical insulator and its electrical conductivity can be restored by adding reducing agents to yield rGO. By incorporating rGO into PCL, the resultant nanocomposites exhibit good electrical conductivity. Sayyar et al. reported that the rGO additions improve electrical conductivity and mechanical strength of PCL [229]. Kumar et al. studied the effect of GO, rGO and amine-functionalized GO (AGO) additions (1, 3 and 5 wt%) on dynamic mechanical behavior, wettability and stem cell response of compression molded PCL disks. They reported that the storage modulus at 25 °C of PCL increased with increasing filler loadings, and this increase was largest for GO followed by AGO and rGO [227]. Thus PCL/rGO nanocomposites had the smallest storage modulus because of the weak interactions between PCL and rGO. The storage modulus of polymers at 25 °C was considered as their elastic modulus. Moreover, GO and AGO additions improved wettability of PCL, i.e., enhanced its hydrophilicity. However, addition of rGO nanofillers with few oxygenated functional groups, increased the hydrophobicity of PCL, leading to an increased contact angle for PCL/rGO composites. 

##### Properties of Porous PCL/GO Scaffolds 

Wan and Chen also fabricated electrospun PCL/0.3 wt% GO scaffold, and reported that the GO filler increases the tensile strength, modulus and energy at break of PCL by 95%, 66% and 416%, respectively. The elastic modulus and tensile strength of electronspun PCL were 10.5 ± 0.92 MPa and 2.37 ± 0.09 MPa, and increased to 17.4 ± 1.25 MPa and 4.61 ± 0.15 MPa, respectively by adding 0.3 wt% GO [228]. The modulus and tensile strength were enhanced by 66% and 95%, respectively. Recently, Song et al. fabricated a series of electrospun PCL/GO mats containing 0.1, 0.3, 0.5, and 1.0 wt% GO [230]. By adding 0.1 wt% and 0.3 wt% GO to PCL, the tensile stress and elastic modulus of the electrospun nanocomposite scaffolds were higher than those of neat PCL. For GO contents ≥0.5 wt%, the values of these tensile parameters decreased dramatically due to the filler aggregation. The enhancement in the mechanical properties of electrospun scaffolds was related to homogeneous dispersion of GO in the PCL matrix, high stiffness of GO, and the interactions between the GO and PCL. Recently, Ramazani and Karimi incorporated both rGO and GO nanofillers into PCL followed by electrospinning [231]. The additions of 0.1 wt% GO and 0.1 wt% rGO to PCL led to an increase in the tensile strength of PCL scaffolds over ~160% and 304% respectively, and an improvement in Young’s modulus over 103% and 163% due to the good dispersion of both nanofillers and their interactions with PCL. As mentioned, rGO is an electrical conductor, so the incorporation of rGO into PCL can create electrically conducting scaffolds. Such conductive scaffolds are beneficial for cell adhesion and proliferation, because they can stimulate an efficient adsorption and deposition of serum proteins on their surfaces, thereby assisting cell attachment and cell growth [232]. 

The small pores formed in electrospun PCL/GO and PCL/rGO scaffolds can limit cell infiltration as mentioned previously. As such, 3D printing is an effective tool to fabricate PCL-based scaffolds with well-defined macropores [233,234,235]. Wang et al. employed AM to fabricate 3D PCL/graphene scaffolds [234,235]. Their graphene sheets were made by dispersing graphite flakes in organic solvents using ultrasonic exfoliation method. This process can produce graphene sheets in large quantities, and the graphene sheets obtained are termed as liquid phase exfoliated (LPE) graphene [236,237]. However, most organic solvents used for the graphite flake exfoliation, e.g., DMF and NMP are highly toxic, and the retention of toxic solvents in the LPE-graphene sheets can induce cytotoxicity. This deleterious effect limits the use of LPE-graphene in biomedical engineering applications. Nevertheless, LPE-graphene additions to 3D printed PCL scaffolds can assist us in understanding pristine graphene-cell interactions in conductive polymer scaffolds. In their study, LPE-graphene additions (0.13, 0.50 and 0.78 wt%) enhance hydrophilicity of 3D printed PCL/graphene scaffolds. The contact angle of PCL scaffold was 96.10°, and decreased to 91.78°, 86.48° and 88.25° by adding 0.13, 0.50 and 0.78 wt% LPE-graphene, respectively. 

#### 3.3.3. PCL/nHA-GO Hybrid Nanocomposite

**Figure 23:**
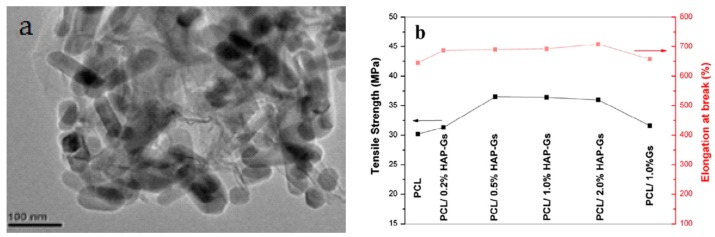
(**a**) TEM image of nHA-Gs showing the attachment of nHA on graphene. (**b**) Tensile strength and elongation at break of PCL and its nanocomposites [238]. Reproduced with permission from Elsevier, published by Elsevier, 2017.

Zhou et al. synthesized nHA-GO hybrid through hydrothermal process, and then incorporated 0.2%, 0.5%, 1% and 2% nHA-GO into PCL using solvent casting [238]. Figure 23a shows that nHA rods are densely anchored on the surface of graphene sheet due to the self-assembly mechanism [130]. This leads to strong interactions between nHA and GO. Moreover, GO converts to rGO or graphene sheets (Gs) during hydrothermal treatment as revealed by the FTIR results. As such, oxygenated groups of GO are removed to a large extent by hydrothermal treatment. Accordingly, the tensile properties of PCL/nHA-GO hybrids are markedly improved due to synergistic effects between the fillers. For comparison, GO is also hydrothermally treated, mixed with PCL, followed by solvent casting to give PCL/1 wt% Gs. The tensile strength and tensile elongation of the PCL/nHA-Gs hybrids are higher than those of PCL/1 wt% Gs (Figure 23b). The improvement in the mechanical properties of hybrid nanocomposites can be ascribed to strong interactions between the nHA and Gs, and uniform dispersion of hybrid fillers in the PCL matrix. 

## 4. In Vitro Studies

### 4.1. In Vitro Hydrolytic Degradation

The hydrolytic degradation behavior of aliphatic polyesters and their nanocomposites is typically carried out through immersion in a phosphate buffer saline (PBS) solution at 37 °C [191,239,240,241]. Ege et al. fabricated dense PLGA and PLGA/25 wt% nHA nanocomposite using twin screw extrusion followed by injection molding [239]. Those samples were then immersed in 0.01 M PBS solution at 37 °C. The results showed that PLGA had faster degradation rate followed by PLGA/25 wt% nHA nanocomposite. Huang et al. fabricated PLA/nHA nanocomposite by melt-mixing, and immersed neat PLGA and nanocomposite in PBS with a pH of 7.4 [240]. They reported that both the flexural strength and modulus of neat PLA deteriorate faster than those of the nanocomposite sample after immersion in PBS for up to 20 weeks. Accordingly, the nanocomposite can maintain sufficient mechanical strength for the bone cell adhesion and growth during the early phase of decomposition. Furthermore, released ions from nHA of the PLA/nHA composite can neutralize lactic acid decomposed from PLA during the degradation process. In this respect, the autocatalytic degradation effect of acids in PLA can be minimized substantially due to the nHA addition, thereby improving bioactivity of PLA. Duan et al. demonstrated that the oxygenated groups of GO improved the wettability of PLA/(0.5–2 wt% GO) nanocomposites, thereby accelerating hydrolytic degradation of PLA in deionized water, HCl and NaOH solutions. The higher GO content in the nanocomposites, the greater the hydrolytic degradation rate was [241]. 

**Figure 24:**
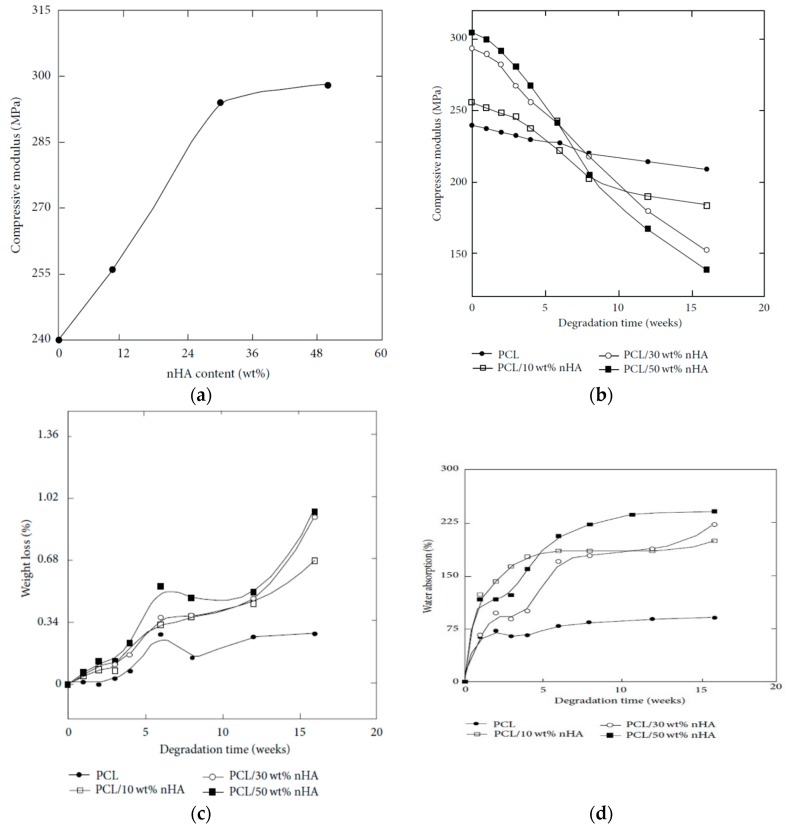
(**a**) Compressive modulus vs. nHA content of PCL/nHA composite scaffolds prior to immersion test. Degradation properties of PCL/nHA composite scaffolds: (**b**) compressive modulus vs. degradation time, (**c**) weight loss vs. degradation time, and (**d**) water uptake vs. degradation time. Reproduced from [242], Hindawi under the Creative Commons Attribution license.

Díaz et al. fabricated PCL/nHA scaffolds with 10 wt%, 30 wt% and 50 wt% nHA by freeze-drying the composite suspensions [242]. Porous scaffolds with porosities of up to 90% were achieved by this process. Pure PCL and nanocomposite scaffolds were immersed in PBS at 37 °C for extended periods of time. Prior to the solution immersion, the compressive modulus of nanocomposite scaffolds increases with increasing nHA content (Figure 24a). Following immersion, compressive modulus of all samples decreases with increasing immersion time. After 5 weeks immersion, the stiffness of the PCL/30 wt% nHA and PCL/50 wt% nHA scaffolds is higher than that of pure PCL and PCL/10 wt% nHA scaffolds (Figure 24b). A similar reducing trend in compressive stress with time is observed for all samples (not shown). Figure 24c reveals that the weight loss of pure PCL scaffold increases slowly with increasing immersion time. During immersion, water penetrates into the amorphous phase of PCL, leading to swelling and chain scission of ester linkage. PCL exhibits a highly crystalline structure and hydrophobicity, thereby hindering water absorption and producing a small weight loss. In contrast, nanocomposite scaffolds undergo larger weight loss, especially those with higher nHA loadings (Figure 24c). Water absorption of the PCL/nHA scaffolds shows a marked increase with immersion time due to the incorporation of hydrophilic nHA fillers (Figure 24d). Apparently, nHA additions accelerate the degradation rate of PCL with poor degradability. Very recently, Sanchez-Gonzalez et al. studied hydrolytic degradation behavior of highly porous PCL and PCL/0.1 wt% rGO scaffold prepared by a phase inversion technique [243]. Both types of scaffolds were immersed in PBS at 37 °C for 12 months. The high internal porosity of the scaffolds facilitated water permeation, leading to cleavage of PCL molecular chains. The presence of rGO slightly accelerated the degradation rate. 

### 4.2. Biomineralization

The bioactivity of porous scaffolds can be assessed through the deposition of apatite layer on their surfaces by incubation in simulated body fluid (SBF) with ionic concentrations similar to those of human blood plasma [244]. Synthetic apatite is a highly bioresorbable material having excellent bioactivity and biocompatibility [245]. Mineralized scaffolds have been found to enhance osteoblastic activities, osteogenic differentiation and bone formation compared with untreated counterparts [12,246,247,248]. The formation of apatite layer on the polymers and composites depends greatly on the ionic concentrations (0.2–2 SBF) of SBF solutions and the compositions of materials [245]. Zhang and Ma reported that apatite crystals coat the PLA surface after incubation in SBF for 15 days. They suggested that that a negatively charged COO^−^ ions derived from PLA attract positive Ca^2+^ ions from SBF, thereby inducing apatite nucleation [249]. However, SBF mineralization test requires prolonged immersion time for depositing apatite layer on the sample surface. Hence the immersion time can be reduced by using higher ion concentrations in SBF. Chen et al. reported that apatite coating can be formed on PLA immersed in supersaturated 5 SBF within 24 h [250]. 

**Figure 25:**
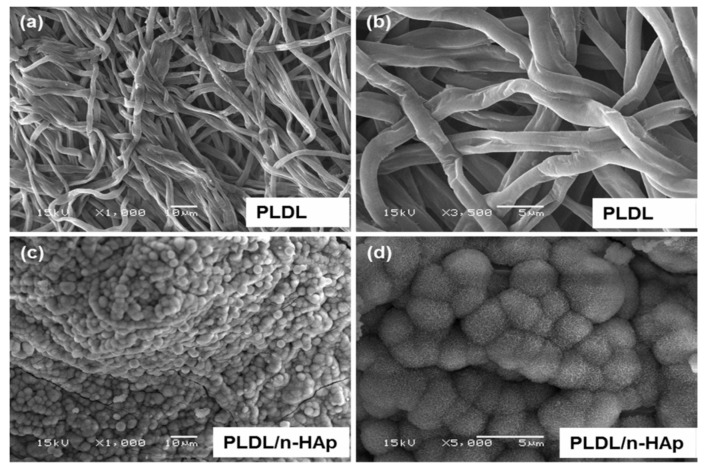
SEM images of electrospun (**a**,**b**) PLDL and (**c**,**d**) PLDL/20 wt% nHA scaffolds after 7 days immersion in 1.5 simulated body fluid (SBF) solution. Reproduced with permission from Springer, published by Springer, 2014.

Tjong and coworkers indicated that nHA fillers of melt-compounded PLA/18 wt% nHA nanocomposite play an important role on the nucleation of apatite layer on its surface upon immersion in SBF solution [55]. The thickness of the apatite layer increases with increasing immersion time. The formation of apatite layer involves the diffusion and precipitation of Ca^2+^ ions from SBF, and their electrostatic interactions with the OH^−^ and PO_4_^3−^ groups of nHA. Rajzer et al. investigated the effect of nHA addition on the apatite layer formation on electrospun poly-l/dl-lactide/20 wt% nHA scaffold upon immersion in SBF solution at 37 °C [251]. Poly-l/dl-lactide (P[L/DL]LA is a copolymer typically used for fracture fixation devices and bone screws. They reported that apatite layer was not deposited on pure PLDL scaffold after 7 days immersion in SBF solution (Figure 25a,b). However, round mineral nodules were deposited on electrospun PLDL/20 wt% nHA fibrous mat, and covered the mat surface completely (Figure 25c,d). These micrographs clearly revealed that nHA fillers acting as effective nucleation sites for mineralization.

**Figure 26:**
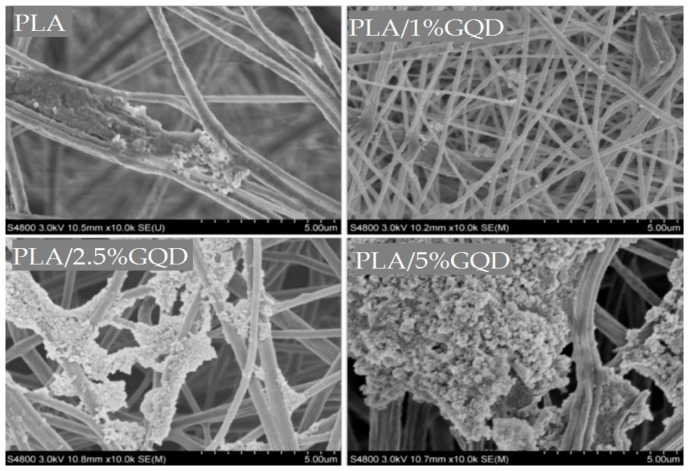
SEM images of electrospun PLA, PLA/1 wt% GQD, PLA/2.5 wt% GQD, and PLA/5 wt% GQD mats after mineralization in SBF for 24 days. Reproduced from [76], MDPI under the Creative Commons Attribution (CC BY) license (http://creativecommons.org/licenses/by/4.0/).

It has been shown that the incorporation of GO and GQD into aliphatic polyesters can also induce the deposition of apatite crystals on their surfaces [76,228]. The oxygenated groups of GO such as carboxyl (−COOH) and hydroxyl (−OH) provide active sites for biomineralization [228,252]. For instance, a thick layer of calcium phosphate mineral can be deposited on electrospun PCL/0.3 wt% GO mat surface by immersion in 10 SBF solution at room temperature for 20 h [228]. Hakkarainen and coworkers demonstrated that the oxygenated groups of GQDs promote the deposition of apatite crystals on electrospun PLA/GQD scaffolds with 2.5 wt% and 5 wt% GQD. Figure 26 shows the SEM images of electrospun PLA and PLA/GQD scaffolds, respectively. From these micrographs, relatively fewer apatite crystals are seen to coat pure PLA scaffold. However, the extent and thickness of apatite crystals grow with increasing GQD contents in the PLA/GQD scaffolds. In particular, higher amounts of mineral deposits can be seen on the PLA/5 wt% GQD surface. Apparently, the additions of GQDs to PLA scaffolds can promote osteoconductivity through stimulating both biomineralization and osteoblastic adhesion/proliferation.

### 4.3. In Vitro Cell Cultivation

#### 4.3.1. Bionanocomposites

Osteoblast differentiation in vitro can be characterized in three stages including cell proliferation, matrix maturation, and matrix mineralization. During proliferation, osteoblasts secrete bone matrix proteins, including collagen type 1 (Col I), osteopontin and fibronectin. Osteoblasts also produce alkaline phosphatase (ALP), i.e., an early marker of osteoblast differentiation, while maturating the ECM with ALP and collagen. Thus, an increased ALP activity is associated with the progressive differentiation of osteoblasts. As recognized, phosphatase is an enzyme that hydrolyzes phosphate group from an organic substrate. *p*-nitrophenyl phosphate is typically used as a substrate to investigate the activity of a phosphatase enzyme. The reaction product, *p*-nitrophenol, turns yellow under alkaline conditions. The product generated from the enzymatic reaction is related to the ALP activity and its absorbance can be examined using a spectrophotometer. The last step proceeds with the matrix mineralization by expressing osteocalcin, which promotes deposition of calcium phosphate mineral [253]. Real-time polymerase chain reaction (RT-PCR) is a powerful tool for revealing the expressions of osteogenic marker genes including ALP, collagen type I (Col I), osteocalcin (OCN) and Runx2 [254,255]. Calcium deposition can be characterized by staining with Alizarin red S dye, and inspection in the phase-contrast microscope. For quantitative analysis, the deposit can be extracted and quantified by a colorimetric assay [256]. 

Lock et al. prepared PLGA/30 wt% nHA bulk nanocomposite by solvent casting. They then seeded hMScs on the nanocomposite with or without BMP-7 derived short peptide [257]. They reported that the PLGA/30 wt% nHA nanocomposite promoted hMSCs adhesion and differentiation with or without osteogenic factors in the culture media. Recently, Kumar et al. cultured hMSCs on compression molded PCL, PCL/GO, PCL/rGO, and PCL/AGO disks containing 1, 3 and 5 wt% fillers. They then analyzed mineral matrix deposition by hMSCs quantitatively using Alizarin red S dye [227]. From their study, PCL/GO and PCL/AGO composites showed significantly higher mineral deposition than neat PCL. The mineral content increased with increasing GO or AGO filler loadings. In particular, PCL/5 wt% AGO exhibited the highest mineral deposition amongst all the samples investigated. They attributed this to the presence of amine groups on AGO surface, leading to enhanced stem cell osteogenesis and mineralization. In other words, there exists a synergistic effect between amine and oxygenated functional groups on the AGO surface in enhancing osteogenesis of hMSCs [227].

**Figure 27:**
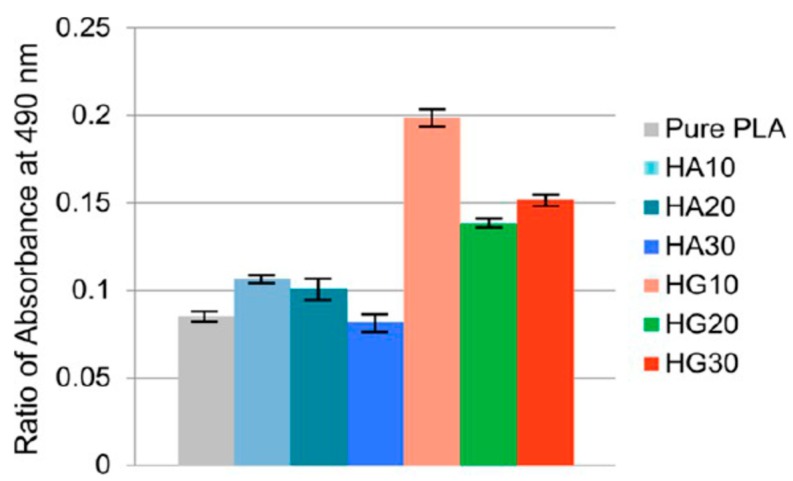
3-(4,5-dimethylthiazol-2-yl)-2,5-diphenyltetrazolium bromide (MTT) assay of MT63 cells cultured on PLA and its nanocomposite films. PLA/nHA films with 10, 20 and 30 wt% nHA were denoted as HA10, HA20, and HA30, respectively. PLA/nHA-GO hybrid films with 10, 20 and 30 wt% nHA-GO were denoted as HG10, HG20, and HG30, respectively. Results are expressed as mean ± standard deviation [201]. Reproduced with permission from the American Chemical Society, published by the American Chemical Society, 2018.

3-(4,5-dimethylthiazol-2-yl)-2,5-diphenyltetrazolium bromide (MTT) assay is typically used to study cell proliferation in vitro. This assay measures cell proliferation or viability through enzymatic conversion of the tetrazolium salt to water insoluble formazan crystals by dehydrogenases occurring in the mitochondria of living cells [258]. Figure 27 shows the MTT results of osteosarcoma cell line (MG-63) cultivated on the PLA/nHA and PLA/nHA-GO nanocomposites with 10–30 wt% filler contents [201]. Apparently, osteoblast cells cultured on pure PLA film exhibit the lowest viability, due to its hydrophobic behavior, and the absence of bioactive sites for the cell adhesion. By contrast, PLA/10 wt% nHA and PLA/20 wt% nHA exhibit higher cell viability or proliferation than pure PLA. At 30 wt% nHA, cell viability drops somewhat as a result of nHA agglomeration at high filler loading. Comparing with PLA/nHA counterparts, PLA/nHA-GO hybrids exhibit higher cell viability, especially for the PLA/1 wt% nHA-GO film. 

#### 4.3.2. Bionanocomposite Scaffolds

##### Bionanocomposite Scaffolds with nHA Fillers

**Figure 28:**
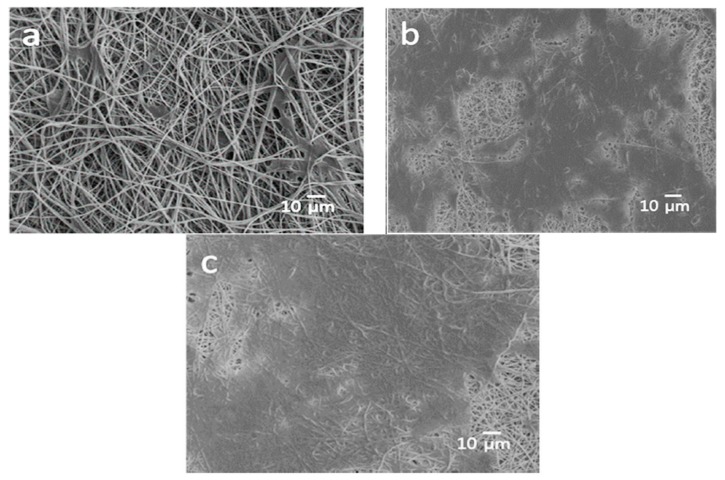
SEM micrographs of osteoblasts seeded onto the surfaces of (**a**) PLA, (**b**) PLA/15 wt% nHA, and (**c**) PLA/15 wt% nHA-1 wt% GO mats. Reprinted from [81], MDPI under the Creative Commons Attribution (CC-BY) license.

**Figure 29:**
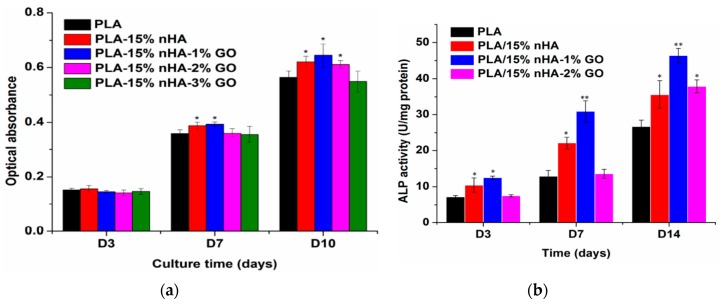
(**a**) MTT assay results of Saos-2 cell cultivated on PLA and its composite fibrous mats for 3, 7 and 10 days. * *p* < 0.05. (**b**) ALP levels of Saos-2 cell cultivated on PLA and its composite fibrous mats for 3, 7 and 14 days. * *p* < 0.05, ** *p* < 0.01. Reprinted from [81], MDPI under the Creative Commons Attribution (CC-BY) license.

Electrospun fibrous scaffolds offer several advantages for bone tissue engineering applications including high porosity, large surface area for cell attachment, and nanofibrous network mimics the structural feature of the natural ECM. Tjong and coworkers have assessed the adhesion and proliferation of osteoblastic Saos-2 cells on electrospun PLA, PLA/15 wt% nHA and PLA/15 wt% nHA-(1–3 wt%)GO mats using MTT assay [81]. The assay measures mitochondrial activity on viable cells by reducing MTT salt to a colored insoluble formazan. Figure 28a–c are typical SEM micrographs showing osteoblasts seeding on electrospun PLA, PLA/15% nHA and PLA/15% nHA-2% GO fibrous mats. Very few osteoblasts are found on the PLA mat surface because it lacks the cell adhesion sites. By contrast, osteoblasts attach, grow and spread over the surface of PLA/15% nHA mat since nHA fillers offer effective seeding sites for osteoblasts. It has been shown that hydrophilic surfaces favor the adsorption of cellular proteins, thereby promoting cell adhesion and growth [248]. nHA and GO nanomaterials can induce hydrophobic PLGA mat to hydrophilic by reducing its water contact angle <90°, especially GO with abundant oxygenated functional groups [123]. In this respect, enhanced bone cell adhesion occurs on fibrous PLA/15% nHA-2% GO mat due to a synergistic effect between nHA and GO fillers in inducing hydrophilicity. The surface of this mat is covered by osteoblasts extensively (Figure 28c). Figure 29a depicts the MTT results showing the viability of Saos-2 cells seeded on electrospun PLA, PLA/15% nHA and PLA/15% nHA-(1–3)% GO fibrous mats. At day 10, PLA/15% nHA, PLA/15% nHA-1% GO and PLA/15% nHA-2% GO fibrous mats display higher cell viability compared to pure PLA mat. The alkaline phosphatase (ALP) activity of representative fibrous mats are depicted in Figure 29b. ALP is an enzyme secreted by osteoblasts, which is identified as an early marker of osteoblast differentiation. Comparing with pure PLA mat, PLA/15% nHA and PLA/15% nHA-1% GO mats exhibit much higher ALP activities at day 7 and day 14. Overall, PLA/15% nHA-1 wt% GO hybrid exhibits highest biocompatibility in vitro.

**Figure 30:**
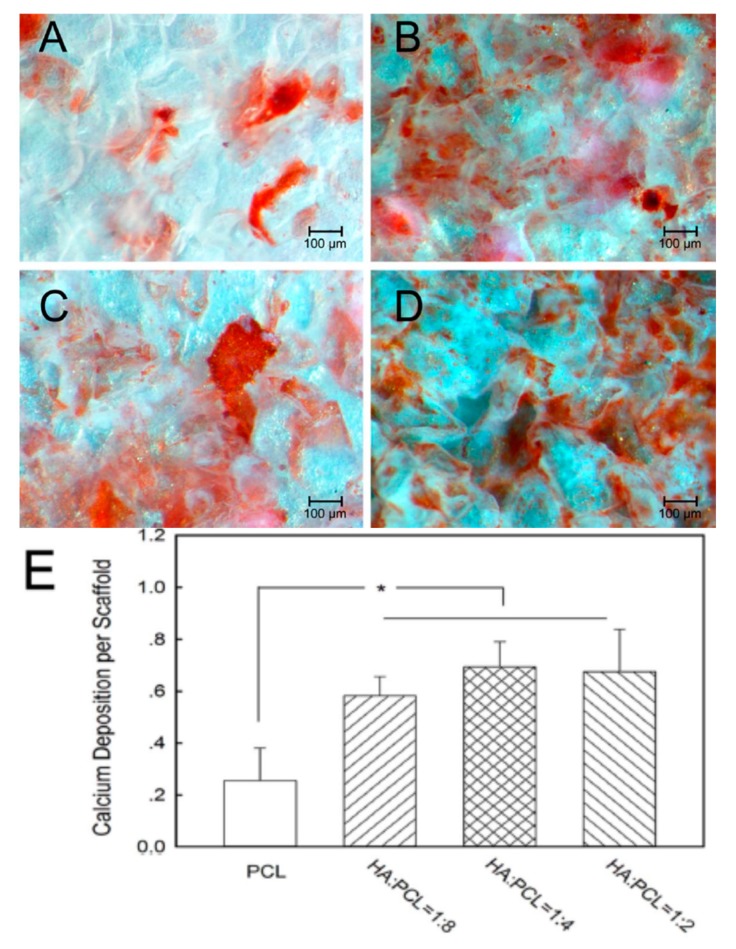
Alizarin red S staining of calcium deposited on PCL/nHA spiral scaffolds. (**A**) Human fetal osteoblasts cultured on neat PCL spiral scaffold, (**B**) PCL/nHA spiral scaffold with nHA:PCL = 1:8, (**C**) PCL/nHA spiral scaffold with nHA:PCL = 1:4 and (**D**) PCL/nHA spiral scaffold with nHA:PCL = 1:2 for 21 days. (**E**) Quantitative analysis of the amount of calcium within each spiral scaffold. Data represent the mean ± standard deviation, *n* = 6. Significant difference between different material groups were denoted as * (*p* < 0.05). Reproduced from [57], PLOS under the Creative Commons Attribution license.

Zhang et al. fabricated PCL/nHA spiral scaffolds with different nHA contents, i.e., nHA: PCL weight ratios of 1:8, 1:4, and 1:2. The pores of those scaffolds were produced by salt leaching. Human fetal osteoblasts (hFOBs) were cultured on porous PCL/nHA scaffolds [57]. They reported that hFOBs can adhere, infiltrate the interconnected pores of spiral scaffolds with high cellular viability. In addition, ALP activity was markedly enhanced with increasing nHA content in the PCL/nHA spiral scaffolds. Furthermore, nHA promoted mineralized matrix formation on the PCL/nHA scaffolds as revealed by Arizarin red S staining (Figure 30). The deposition of calcium by hFOBs in the extracellular matrix is an indicative of osteogenesis and can be considered as a marker for bone regeneration. Morelli et al. reported that electrospun PLA/20 wt% nHA mat induced osteogenic differentiation of hMSCs and osteoclastogenic differentiation as evidenced by high expression of specific gene markers such as osteopontin, osteocalcin, ALP, and receptor activator of nuclear factor kappa B (RANK) [184]. Bone remodeling is a dynamic process involving the resorption of old bone by osteoclasts, and the formation of new bone tissue by osteoblasts. 

##### Bionanocomposite Scaffolds with GO Fillers

**Figure 31:**
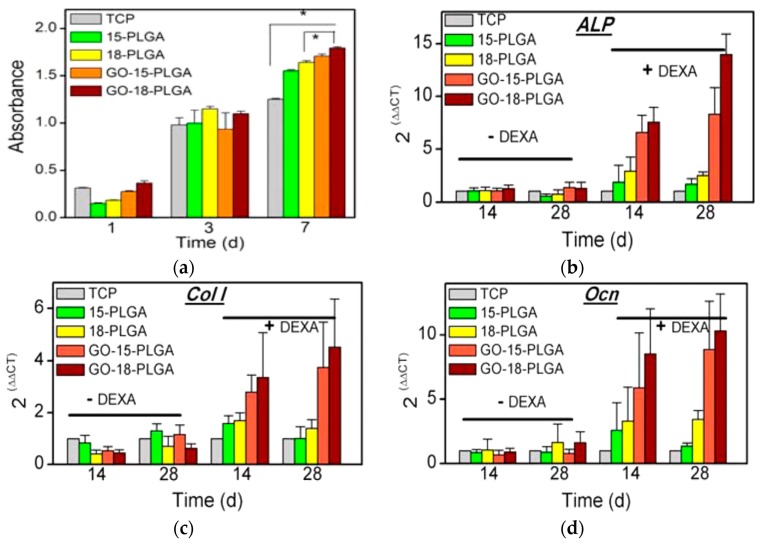
(**a**) MTT assay showing the proliferation of human mesenchymal stem cells (hMSCs) cultured on tissue culture plate (TCP), electrospun PLGA copolymers and their nanocomposite scaffolds with or without dexamethasone (DEXA), for 14 and 28 days, respectively; (* *p* < 0.05). Real time polymerase chain reaction (RT-PCR) analysis of osteogenic marker genes of hMSCs seeded on electrospun PLGA copolymers and their nanocomposite scaffolds with or without DEXA, for 14 and 28 days, respectively: (**b**) Alkaline phosphatase (ALP), (**c**) Collagen I and (**d**) Oesteocalcin (OCN) [78]. Reproduced with permission from the American Chemical Society, published by the American Chemical Society, 2015.

Graphene and its derivatives have been reported to be very effective in promoting osteogenic differentiation of mesenchymal stem cells [78,82,83,84,85,120,174,175,176,259]. Liang et al. reported that the addition of 1.5 wt% GO to PLGA/nHA scaffold enhances the proliferation of mouse osteoblasts (MC3T3-E1) [120]. Luo et al. studied the effect of GO additions on the proliferation and osteogenetic differentiation of hMCSs on electrospun PLGA mats [78]. They reported that GO nanofillers play two crucial roles in the fibrous mats, i.e., enhance the hydrophilic performance, and accelerate hMSCs adhesion, proliferation and osteogenic differentiation. The MTT assay results showing the proliferation of MSCs cultured on the 15 wt% PLGA, 18 wt% PLGA and their nanocomposite fibrous mats for different time periods are given in Figure 31a. By adding 1 wt% GO to both PLGA suspensions, the resulting composite fibrous mats display enhanced adhesion and proliferation of MSCs. Figure 31b–d shows the RT-PCR assay results for these fibrous mats with or without dexamethasone (DEXA). Differentiation of hMSCs to osteoblastic lineages in vitro is associated with their ability to express Col I and ALP. Col I is expressed by osteoblasts during the initial period of proliferation and ECM synthesis, while ALP is expressed during the post proliferative period of matrix maturation [260,261,262,263]. Osteogenic differentiation of hMSCs in vitro is greatly enhanced by DEXA. In the presence of DEXA, the amounts of ALP, OCN and Col I extracted from hMSCs cultured on the 15-PLA/1% GO and 18-PLA/1% GO nanocomposite mats are substantially higher than those cultured on the 15-PLGA and 18-PLGA scaffolds. This indicates that GO nanofillers are very effective to promote stem cells differentiation. In general, many in vitro stem cell experiments often use DEXA, ascorbic acid and β-glycerophosphate to enhance osteogenic expression levels [264,265]. DEXA induces MSCs differentiation into osteoblasts by activating Runx2, ascorbic acid increases the secretion of Col I, and β-glycerophosphate serves as a source for the phosphate in hydroxyapatite [265]. 

**Figure 32:**
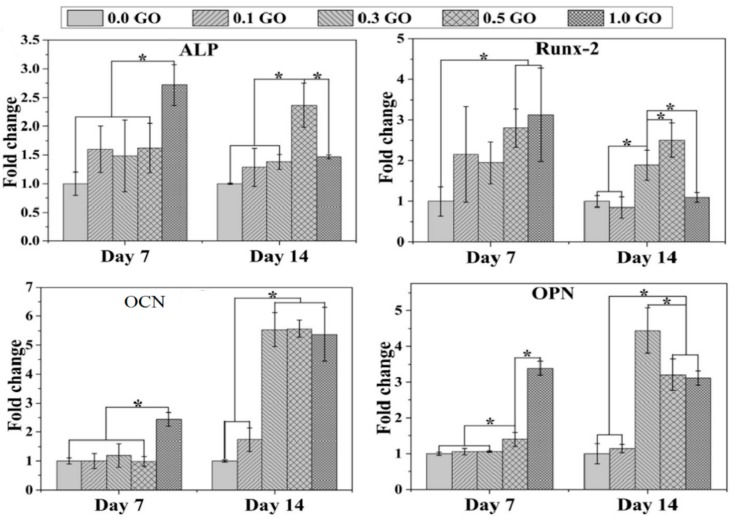
The gene expression analysis of ALP, Runx2, OCN, and OPN for mouse marrow mesenchymal stem cells (mMSCs) differentiated on electrospun PCL/GO nanocomposite scaffolds with different GO contents at day 7 and day 14, respectively. * represents significant difference between different material groups [230]. Reproduced with permission from Elsevier, published by Elsevier, 2015.

As aforementioned, the oxygenated groups of GO provide active sites for depositing calcium phosphate mineral on electrospun PCL/0.3 wt% GO mat surface by immersion in 10 SBF solution [228]. Fu et al. reported that the inclusion of 2 wt% GO to PCL lead to calcium deposition by MC3T3-E1 mouse pre-osteoblasts on electrospun PCL/GO mat surface as revealed by Alizarin red S staining [266]. Song et al. studied the effect of different GO filler contents (0.1, 0.3, 0.5 and 1.0 wt%) in electrospun PCL/GO scaffolds on the proliferation and differentiation of mouse marrow mesenchymal stem cells (mMSCs) [230]. At day 7, the gene expression levels of ALP and Runx2 increase with increasing GO contents during the initial stage of cell differentiation. So the PCL/1 wt% GO mat exhibits the highest level of gene expressions of ALP and Runx2 (Figure 32). Runx2 is an osteogenesis specific transcription factor, which is crucial for the differentiation and maturation of osteoblasts. At day 14, these gene levels decrease as the cells proceed to a late stage of differentiation. However, OCN gene level of the PCL/GO mats with GO contents ≥0.3 wt% increases markedly at day 14. OCN is a marker of bone formation and secreted by osteoblasts, and it involves in the late stage of mineralization. From the literature, osteogenic differentiation of mMSCs in vitro proceeds into several stages. The first stage consists of enhanced cell adhesion and proliferation (days 1 to 4), followed by early cell differentiation as demonstrated by enhanced ALP activity (days 5 to 14). Thereafter, ALP level begins to decline, and the mineralization produces high levels of OCN and osteopontin (OPN), followed by Ca and phosphate deposition (days 14 to 28) [267,268]. OPN is an integrin-binding glycoprotein present in bone matrix, which is important for bone remodeling and immune system signaling. In general, the cell differentiation periods varies greatly from one to another, depending upon the cell culture conditions. Apart from the osteogenic differentiation of MSCs, electrospun PCL/GO nanofibers have been reported to guide the differentiation of neural stem cells into mature oligodendrocytes, i.e., myelinating cells of the central nervous system [269]. 

**Figure 33:**
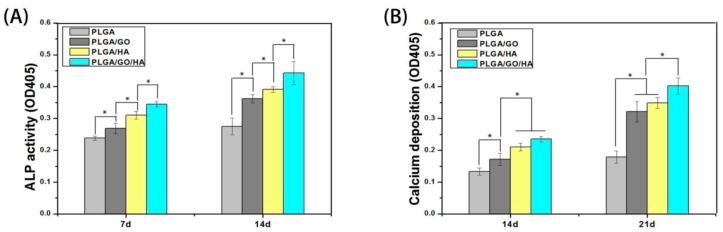
(**A**) ALP activities of MC3T3-E1 cells cultivated on electrospun PLGA, PLGA/2 wt% GO, PLGA/10 wt% nHA and PLGA/10 wt% nHA-2 wt% GO mats for 7 and 14 days. (**B**) Calcium deposition after cell culturing on these nanofibrous mats for 14 and 21 days. * represents significant difference between different material groups; *p* < 0.05, *n* = 4. Reproduced from [123], PLOS under the Creative Commons Attribution license.

**Figure 34:**
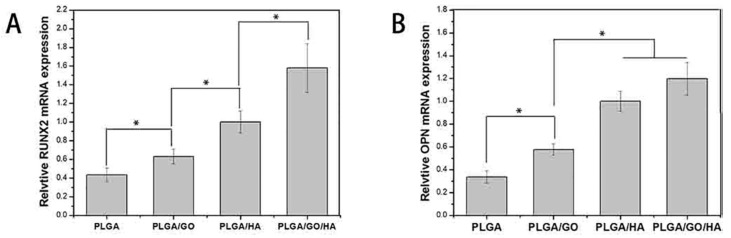
Quantitative RT-PCR analysis of osteogenic gene expressions: (**A**) Runx2 and (**B**) OPN for MC3T3-E1 cells cultured on PLGA-based fibrous mats for 7days; * represents significant difference between different material groups; *p* < 0.05, *n* = 3. Reproduced from [123], PLOS under the Creative Commons Attribution license.

The cell differentiation on the hybrid composite scaffolds is now considered. Fu et al. conducted a comprehensive study on the structure, tensile behavior and wettability of electrospun PLGA, PLGA/10 wt% nHA, PLGA/2 wt% GO and PLGA/10 wt% nHA-2 wt% GO scaffolds. Overall, hybrid PLGA/10 wt% nHA-2 wt% GO scaffold exhibits the highest tensile strength and excellent hydrophilicity compared to pure PLGA, PLGA/10 wt% nHA, and PLGA/2 wt% GO mats [123]. Alizarin red S staining was used to assess the calcium deposition by MC3T3-E1 cells cultured on all fibrous scaffolds for 14 and 21 days. They demonstrated that the PLGA/10 wt% nHA-2 wt% GO hybrid scaffold greatly enhanced the adhesion and proliferation of MCET3-E1 cells, and effectively promoted their ALP activity and mineral deposition (Figure 33), as well as increased osteogenic gene expressions of Runx2 and OPN (Figure 34). Apparently, PLGA/10 wt% nHA-2 wt% GO scaffold exhibited the highest gene expression levels of Runx2 and OPN as well as calcium deposition compared to other nanofibrous mats. These results implied that hybridization of GO and nHA nanofillers is beneficial in enhancing osteodifferentiation of MC3T3-E1 cells. 

**Figure 35:**
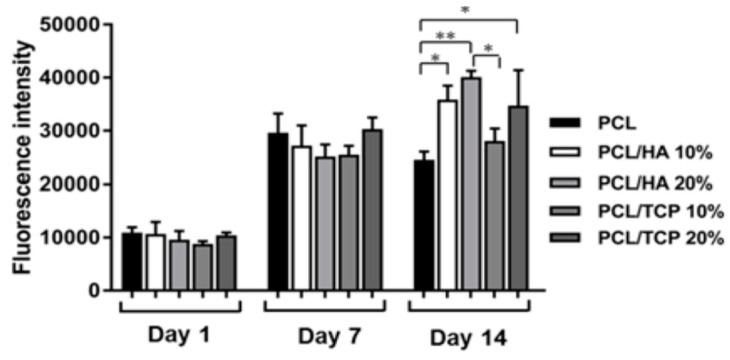
Fluorescent intensity of human adipose derived stem cells (hADSCs) seeded on the PCL and its nanocomposite scaffolds; * *p* < 0.05 and ** *p* < 0.01. Reproduced from [217], MDPI under the Creative Commons Attribution (CC BY) license (http://creativecommons.org/licenses/by/4.0/).

**Figure 36:**
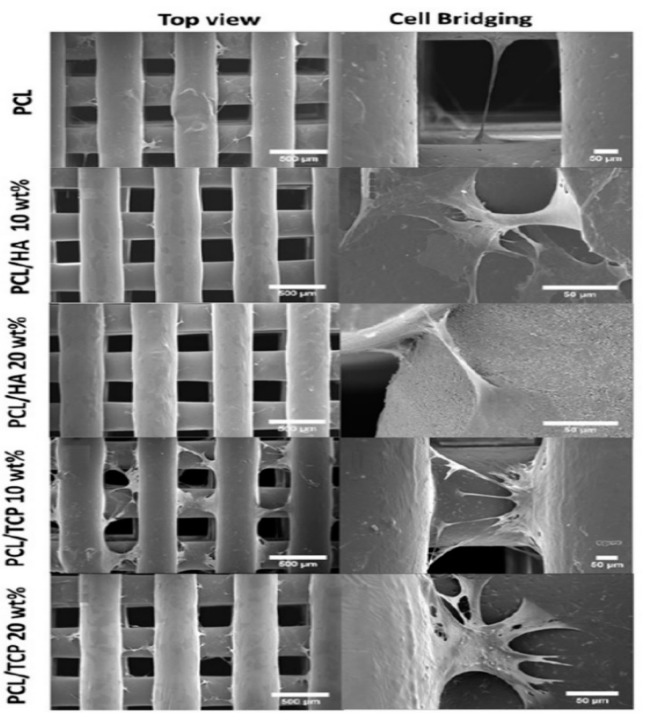
SEM images showing cell attachment and cell bridging on the PCL, PCL/10 wt% nHA, PCL/20 wt% nHA, PCL/10 wt% TCP, and PCL/20 wt% TCP scaffolds, respectively. Reproduced from [217], MDPI under the Creative Commons Attribution (CC BY) license (http://creativecommons.org/licenses/by/4.0/).

As mentioned, AM technology enables the creation of bionanocomposite scaffolds with precisely controlled pore size, porosity and interconnecting pore network. By incorporating those pore factors into design models, the constructed 3D scaffolds allow efficient cell penetration, tissue ingrowth and vascularization [270,271,272]. Huang et al. employed an extrusion-based AM system to print 3D PCL, PCL/10 wt% nHA, PCL/20 wt% nHA, PCL/10 wt% TCP and PCL/20 wt% TCP scaffolds [217]. The cell viability of human adipose derived stem cells (hADSCs) seeded on such nanocomposite scaffolds were assessed by Alamar Blue assay (Figure 35). At day 14, PCL/10 wt% nHA and PCL/20 wt% nHA scaffolds, especially the latter exhibited higher fluorescent intensity compared to pure PCL and PCL/TCP scaffolds. This led to an increase in cellular proliferation on their surfaces, implying good biocompatibility of PCL/nHA scaffolds. Figure 36 depicts the cell morphology of PCL, PCL/10 wt% nHA, PCL/20 wt% nHA, PCL/10 wt% TCP and PCL/20 wt% TCP scaffolds at day 14. It can be seen that very few cells adhere and migrate on 3D PCL scaffold due to its poor bioactivity. Nevertheless, these cells tend to bridge the polymer layers and filaments of the scaffold. For the PCL/10 wt% nHA, PCL/20 wt% nHA, PCL/10 wt% TCP and PCL/20 wt% TCP scaffolds, adipose stem cells tend to link each other to form dense and continuous cell sheets. Those cell sheets also bridge across the filaments, demonstrating that the scaffolds can support the adhesion and growth of stem cells. It is noted that that same research group also fabricated 3D PCL/LPE-graphene scaffolds with a 0°/90° lay-down pattern using an extrusion-based AM system [234,235]. Similarly, hADSC cell sheets are seen to bridge orthogonal scaffold filaments despite the presence of residual organic solvents in LPE-graphene. Moreover, LPE-graphene is beneficial in enhancing hydrophilicity of 3D printed PCL/graphene scaffolds as mentioned previously. This demonstrates that LPE-graphene is very effective to promote the attachment and proliferation of adipose stem cells. 

Xia et al. investigated the bioactivity and biocompatibility of SLS-fabricated PCL/(5–15 wt%) nHA scaffolds [215]. They reported that nHA fillers promote the formation of apatite crystals on the PCL/nHA scaffolds upon immersion in the SBF solution at 37 °C. Furthermore, the inclusion of nHA to PCL was beneficial in enhancing adhesion, proliferation of hMSCs on the basis of CCK-8 assay. In particular, PCL/15 wt% nHA exhibited the highest cell proliferation, thus it offered an excellent microenvironment for the growth of hMSCs. Furthermore, PCL/15 wt% nHA also exhibited the highest level of ALP expression. Alizarin red S staining revealed that calcium deposition of hMSCs cells cultured on the PCL/nHA scaffolds is more obvious than that on PCL scaffold, especially for the PCL/15 wt% nHA. This implied that the PCL/nHA scaffolds facilitate more hMSC mineralization than their PCL counterpart.

## 5. In Vivo Animal Models

Comparing with in vitro cell cultivation, fewer information is available in the literature relating in vivo animal models of porous scaffolds prepared from aliphatic polyesters reinforced with nHA and/or GO as well as their hybrid nanofillers. Pure PLA and PCL polymers are hydrophobic, so they lack bioactive sites for the cell adhesion. In the absence of nHA or GO, pure aliphatic polyester scaffolds must be seeded with bioactive molecules or growth factors followed by implanting into bone defects of animal models. For instance, Zhang et al. loaded simvastatin (SIM) into 3D PLGA scaffolds fabricated by the FDM process; collagen was also incorporated into the scaffolds for surface activation [273]. They reported that such PLGA/Col/SIM scaffolds can induce new bone formation at the defects created at the femurs of Sprague-Dawley rats. Williams et al. carried out in vivo study of SLS-processed PCL scaffolds in mice. The scaffolds were seeded with BMP-7 transduced fibroblasts, and implanted subcutaneously into mice for inducing ingrowth of bone tissue [214].

Moncal et al. prepared a microcomposite ink solution composed of PLGA, PCL, and HA microparticles with a ratio of 4.5:4.5:1, and 3D printed it into porous constructs [149]. The printed constructs were then implanted into rat calvarial defects. The results showed that 3D-printed PLGA-PCL/HA microcomposite constructs promoted bone regeneration due to the formation of mineralized bone tissue and blood vessel. Very recently, Nguyen et al. implanted solvent-cast PLA/30 wt% d-nHA nanocomposite into dog femurs [274]. Magnesium and zinc-doped nanohydroxyapatite (d-nHA) was used as the filler material. They reported that the nanocomposite did not induce osteitis, osteomyelitis or structural abnormalities after implantation for three months. The histological and x-ray image showed that the implant material promoted new bone growth effectively. Zhang et al. combined the phase inversion and porogen leaching to fabricate PLGA/10 wt% nHA scaffold. This scaffold was then coated with polydopamine (PDA), followed by treated with either BMP-2 or insulin-like growth factor 1 (IGF-1) [275]. Both the BMP-2 and IGF-1 immobilized PLGA/10 wt% nHA scaffolds were finally implanted into the rabbit radius defects, leading to the highest osteoconductivity and excellent bone healing capacity.

**Figure 37:**
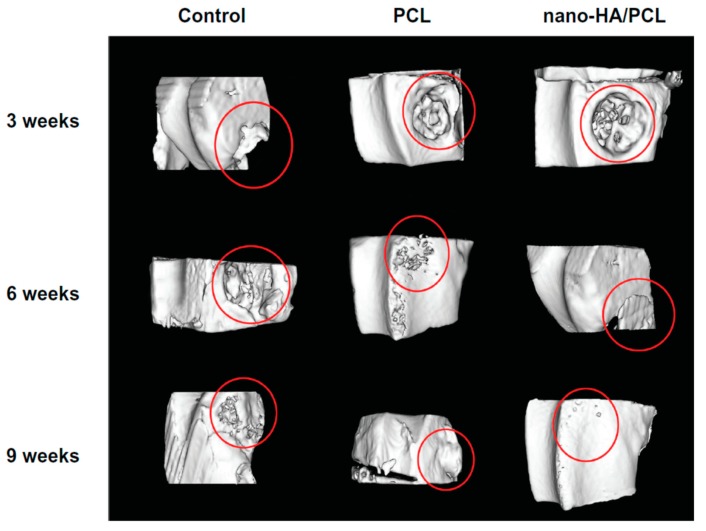
MicroCT reconstruction images of the PCL and PCL/15 wt% nHA scaffolds implanted into rabbit distal femur for 3, 6, and 9 weeks. Circle denotes implanted region. Reproduced from [215], Dove Medical Press under the Creative Commons Attribution—Non Commercial (unported, v3.0) license.

Xia et al. reported that SLS-processed PCL/15 wt% nHA scaffold had the highest cell proliferation, highest ALP activity, and the most obvious Alizarin Red S staining [215]. Thus, PCL/15 wt% nHA and pure 3D PCL scaffolds were implanted separately into New Zealand rabbits to study osteogenesis in vivo. Bone defects were created at the distal femur of rabbits, and all scaffolds were then implanted into the defects. The rabbits were then scanned in a microcomputed tomography (microCT) system at time points of 3, 6, and 9 weeks (Figure 37). Following week 3 surgery, the scaffolds in bone defects remained visible in the control, implanted PCL and PCL/15 wt% nHA groups of rabbits. The microCT images revealed no bone formation within the muscles. At week 6, the PCL/15 wt% nHA scaffold was almost filled with newly formed bone tissue. At week 9, it showed nearly complete degradation, and extensive bone ingrowth throughout the entire defect. Apparently, nHA filler is beneficial in accelerating the degradation rate of PCL under in vivo and in vitro conditions [242]. In addition, nHA also promotes new bone growth in vivo. 

**Figure 38:**
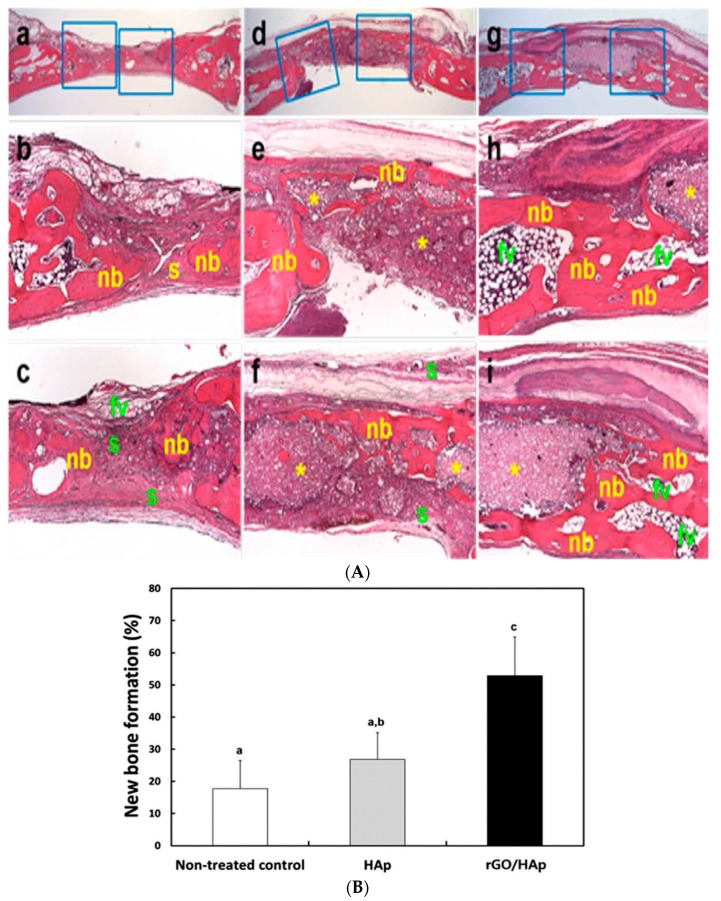
Histological examinations. (**A**) Optical images from hematoxylin-eosin (HE) staining. (**a**–**c**): non-treated control, (**d**–**f**): the defects treated with HA grafts, and (**g**–**i**): the defects treated with rGO/HA grafts. Original magnifications: ×12.5 in (**a**,**d**,**g**), and ×50 in the others. Symbols: nb, new bone; s, soft tissue; fv, fibrovascular tissue; *, graft materials. (**B**) New bone formation (%).The data is expressed as the mean ± SD (*n* = 4) based on at least duplicate observations. The small letters denote a significant difference between non-treated control and the defects implanted with HA and rGO/HA grafts, *p* < 0.05. Reproduced from [129], Nature Research under the Creative Commons Attribution license (http://creativecommons.org/licenses/by/4.0/).

At present, there is no information relating in vivo animal models of aliphatic polyesters reinforced with GO and its derivatives. For other copolyester biomaterial, Zhou et al. conduced in vivo bone repair study of electrospun poly(3-hydroxybutyrate-*co*-4-hydroxybutyrate)/GO scaffold. The P3-4HB/GO scaffold was implanted into full-thickness rat calvarial defects [276]. They reported that GO nanofillers facilitate new bone generation at rat bone defects. Lee et al. fabricated hybrid rGO/HA grafts, and then implanted into calvarial bone defects of 12–13 week-old male New Zealand white rabbits [129]. In their study, full-thickness calvarial bone defects (6 mm in diameter and 2.5 mm in depth) were made in the rabbits by trephining. Hybrid rGO/HA grafts were then seeded into rabbit calvarial bones, while unseeded bone defects in other rabbits without the grafts were used as the control model. For the purposes of comparison, HA grafts were also seeded into the calvarial bones. They reported that hybrid rGO/HA grafts were very effective for promoting new bone formation in rabbit calvarial defects when compared with HA grafts (Figure 38A). For the control model, the non-treated defects were filled with thin and loose connective tissues with minimal new bone formation after four weeks of surgery (left figure panel). By contrast, the defects treated with HA grafts were filled with dense connective tissue (center panel). Moreover, several newly formed bone were observed in the defects treated with hybrid rGO/HA grafts, demonstrating the accelerated bone remodeling process (right panel). The histometric analysis showed that the new bone density in hybrid rGO/HA grafts was significantly higher than that in the other groups (Figure 38B). In this respect, it is expected that hybrid PLA/rGO/HA nanocomposite scaffolds would also promote new bone formation upon implantation into animal models. More future work is needed to elucidate this beneficial effect in degradable polymer bionanocomposites. Very recently, Peng et al. fabricated non-degradable PEEK/nHA scaffolds reinforced with 1 wt% GO through FDM process [277]. It is noted that PEEK is not a polyester. The scaffolds were then implanted into rabbit radial bone defects. They reported that new bone formed throughout the scaffolds after implantation for 60 days. 

Despite osteogenesis induced by the GO fillers of P3-4HB/GO scaffold in vivo [276], the biosafety of GO is an essential concern for its bone tissue engineering applications. After bone regeneration, biodegradable aliphatic polyesters degrade with time, so GO nanofillers are left behind in the host tissue. GO nanofillers are difficult to remove from the host tissue due to their nanoscale dimension. In this respect, the cytotoxicity of GO becomes a major issue for its clinical use. The biocompatibility and cytotoxicity of standalone GO in vivo have been widely studied [66,277,278,279,280,281,282,283]. Conflicting results were reported relating cellular responses to standalone GO and its derivatives in vivo. For example, Ali-Boucetta et al. found that GO did not cause inflammation and granuloma formation in mice after intraperitoneal injection [279]. Yang et al. indicated that GO retained in the mice tissues for a long period of time following intraperitoneal injection, its toxicity to the mice was insignificant [280]. However, other workers reported that GOs can induce cytotoxicity in animal models, and the cytotoxic effect was dependent upon the dose and size of GOs [281,282,283]. Till to present, there exists no reports in the literature on the biological effects and cytotoxicity induced by the GO nanofillers of aliphatic polyester scaffolds. Recently, Kanayama et al. coated collagen scaffolds with GO and rGO, and then subcutaneously implanted into male Wistar rats [284]. From the histological examination of rat tissues, they reported that inflammatory cells such as neutrophils and lymphocytes were rarely seen. Instead, numerous macrophages appeared and ingested GO and rGO, forming giant cells. Therefore, it needs a thorough in vivo biocompatibility and cytotoxicity assessment of aliphatic polyester/GO scaffolds for bone tissue engineering applications. 

## 6. Conclusions

We have presented current trends on the development of aliphatic polyester scaffolds reinforced with nHA and/or GO and its derivatives, especially in the past four years. The fabrication, structure, mechanical behavior, hydrolytic degradation, biomineralization, in vitro cell cultivation and in vivo animal model studies of such scaffolds are systematically reviewed. Nanohydroxyaptite and GO are excellent reinforcements for aliphatic polyester scaffolds that provide mechanical support for the proliferation and cell ingrowth during tissue regeneration. Furthermore, nHA and GO nanofillers can increase bioactivity, osteoblastic cell adhesion and proliferation as well as osteogenic differentiation of MSCs. Significant enhancements in the mechanical properties, bioactivity and biocompatibility of aliphatic polyester composite scaffolds can be achieved by hybridizing nHA and GO nanofillers. 

Aliphatic polyester nanocomposite scaffolds can be fabricated using conventional processing techniques such as solvent casting/particulate leaching, TIPS and electrospinning. These techniques require the use of organic solvents that pose serious risk to the environment and to human health. The major drawbacks of porogen leaching are poor control of the pore geometry and pore interconnectivity, and the difficulty of removing porogens deep inside the polymer matrix of thick scaffolds. The TIPS technique can result in low connectivity and difficulty in controlling the pore size. 

Electrospun aliphatic polyester nanocomposite scaffolds reinforced with nHA, and GO as well as nHA/GO nanofillers have been shown to exhibit excellent bioactivity and biocompatibility. However, their fined pores hinder in vitro cell penetration, in vivo tissue ingrowth and vascularization. In certain case, the average pore size of PCL/nHA mats falls into about 2 µm range [214]. Osteoblastic and tissue ingrowth as well as their associated mineralization processes require bone scaffolds with large macropores (200–400 μm). As a result, osteoblasts can only attach on the electrospun mat surface, so the tissue growth is limited to the periphery of scaffold. In this respect, 3D AM techniques appear to be very effective for making bone scaffolds with large macropores. In particular, FDM process has emerged as an effective tool to fabricate 3D bone scaffolds with precisely controlled pore size, pore shape and porosity. Apart from the macropores, micropores in the filaments of FDM-processed aliphatic polyester scaffolds provide a better environment for the cell attachment, proliferation and function. Moreover, advanced AM techniques can design and manufacture biomimetic tissue scaffolds with much higher mechanical strength and stiffness than conventional fabrication processes. 

In the context of tissue engineering, bioinks consisting of nanocomposite components and biological cells appears to be attractive for printing vascularized constructs for bone tissue engineering applications. Research on novel compatible biomaterials for bioprinting of vascularized constructs is in the embryonic stage [155,156,157,285]. It is anticipated that the development of vascularized networks can enhance new tissue formation and remodeling, leading to host tissue integration upon implantation. 

Finally, we must consider whether or not GO and its derivatives caused toxicity to human tissues. Conflicting reports in the literature suggests that GO and its derivatives can be either biocompatible or toxic to mammalian cells [66,278,279,280,281,282,283]. Graphene oxide is generally synthesized from modified Hummers process by immersing graphite flakes in strong oxidants under sonication. However, different types and concentrations of oxidants, and various oxidation times are used by the researchers for synthesizing GO. These lead to the as-synthesized GO having different physicochemical properties in terms of the layer numbers, lateral sizes, impurity levels and the C/O ratios [97,100,103]. The physicochemical properties of GO significantly affect its biocompatibility, cytotoxicity and biodistribution in vivo. Thus a standard protocol for synthesizing GO is needed to avoid these issues. We speculate that standalone GO dispersed in the solution suspension or cell culture medium may induce cytotoxicity by penetrating into cytoplasm. However, GO fillers of polymer nanocomposite scaffolds are firmly bonded to the polymeric matrix materials. As such, GO nanofillers cannot detach from the polymer matrix and penetrate into cytoplasm, and their cytotoxic effects are minimized or eliminated accordingly. Limited in vivo study reveals that GO nanofillers of non-degradable PEEK-nHA/1 wt% GO scaffold are nontoxic to rabbit bone tissue [277]. For porous scaffolds with aliphatic polyester nanocomposites, the polymer matrix must degrade in order to be finally replaced by the bone tissue. So GOs may release from the scaffolds, and enter the host tissue. Till to present, there is no information available in the literature on in vivo cytotoxicity of porous scaffolds prepared from aliphatic polyesters with GO nanofillers and their derivatives. Thus in vivo animal models must be performed by the researchers in order to ensure the safe use of such novel bionanocomposite scaffolds for bone tissue engineering applications. 

## Figures and Tables

**Table 1:** Mean structural and mechanical properties of porous scaffolds derived from aliphatic polyesters and their nanocomposites reinforced with nHA or GO nanofillers.

Specimen Composition, wt%	Fabrication Route	Porosity, %	Pore Size, µm	Fiber Diameter, nm	Mechanical Test Method	Modulus, MPa	Strength, MPa	Elongation, %	Ref.
***PLA-Based Scaffolds***									
PLA	SC/PL	92.0	NA	None	Compression	4.7	0.29	NA	[61]
PLA/10% nHA	SC/PL	91.4	NA	None	Compression	5.0	0.32	NA	[61]
PLA/20% nHA	SC/PL	90.6	NA	None	Compression	6.4	0.35	NA	[61]
PLA/50% nHA	SC/PL	86.5	NA	None	Compression	9.8	0.44	NA	[61]
PLA	TIPS	93.0	50–100	None	Compression	4.3	NA	NA	[60]
PLA/10% nHA	TIPS	92.8	50–100	None	Compression	4.9	NA	NA	[60]
PLA/30% nHA	TIPS	92.3	50–100	None	Compression	7.8	NA	NA	[60]
PLA/50% nHA	TIPS	91.8	50–100	None	Compression	8.3	NA	NA	[60]
PLA	TIPS	87.4	175	None	Compression	2.4	1.79	NA	[179]
PLA/50% nHA	TIPS	85.1	175	None	Compression	14.9	8.67	NA	[179]
PLA	ES	NA	4.51	365	Tension	0.42	0.063	27	[182]
PLA/5% nHA	ES	NA	1.32	255	Tension	1.80	0.157	30	[182]
PLA/20% nHA	ES	NA	0.53	135	Tension	4.71	0.262	36	[182]
PLA	LAM	55.3	271	None	Tension; Bending	2.8 (B)	42.5 (T); 122.8 (B)	NA	[190]
PLA/10% nHA	LAM	68.5	336	None	Tension; Bending	3.1 (B)	38.6 (T); 131.9 (B)	NA	[190]
PLA/20% nHA	LAM	85.1	392	None	Tension; Bending	3.5 (B)	35.1 (T); 138.6 (B)	NA	[190]
PLA/30% nHA	LAM	76.3	354	None	Tension; Bending	3.8 (B)	29.2 (T); 119.1 (B)	NA	[190]
PLA/40% nHA	LAM	72.2	318	None	Tension; Bending	3.9 (B)	23.2 (T); 112.5 (B)	NA	[190]
PLA	ES	NA	NA	839	Tension	NA	2.1	97	[200]
PLA/1% GO	ES	NA	NA	706	Tension	NA	2.9	85	[200]
PLA/2% GO	ES	NA	NA	863	Tension	NA	3.5	87	[200]
PLA/1% GO-*g*-PEG	ES	NA	NA	593	Tension	NA	3.9	95	[200]
PLA/2% GO-*g*-PEG	ES	NA	NA	761	Tension	NA	4.5	83	[200]
PLA/5% GO-*g*-PEG	ES	NA	NA	685	Tension	NA	5.2	84	[200]
PLA	ES	70.5	NA	786	Tension	8.58	0.27	NA	[81]
PLA/15% nHA	ES	74.5	NA	563	Tension	9.88	0.41	NA	[81]
PLA/15% nHA-1% GO	ES	75.6	NA	516	Tension	12.69	0.47	NA	[81]
PLA/15% nHA-2% GO	ES	76.2	NA	502	Tension	16.73	0.57	NA	[81]
PLA/15% nHA-3% GO	ES	77.9	NA	412	Tension	8.10	0.38	NA	[81]
***PCL-Based Scaffolds***									
PCL	ES	NA	1.94	393	Tension	NA	12.3	380	[213]
PCL/30% nHA	ES	NA	2.19	317	Tension	NA	85.17	530	[213]
PCL/60% nHA	ES	NA	2.30	332	Tension	NA	158.1	564	[213]
PCL	SLS	63.1	1750 × 1750	NA	Compression	65	3.2	NA	[214]
PCL	SLS	79.0	2000 × 2500	NA	Compression	54	2.0	NA	[214]
PCL	SLS	78.54	NA	NA	Compression	NA	1.38	NA	[215]
PCL/10% nHA	SLS	72.06	NA	NA	Compression	NA	2.67	NA	[215]
PCL/15% nHA	SLS	70.31	NA	NA	Compression	NA	3.17	NA	[215]
PCL	ES	94.1	NA	543	Tension	10.5	2.37	110	[228]
PCL/0.3% GO	ES	94.6	NA	640	Tension	17.4	4.61	275	[228]
Trabecular bone; dried defatted	NA	NA	NA	NA	Compression	1.1–139	0.3–7.0	NA	[15]
Trabecular bone; fresh frozen	NA	NA	NA	NA	Compression	1.4–79	1.5–45	NA	[15]

Abbreviations: B, three-point bending; ES, electrospinning; LAM, low temperature additive manufacturing; NA, not available; PL, porogen leaching; SC, solvent casting; SLS, selective laser sintering; T, Tension; TIPS, thermally induced phase separation.
